# Rastreamento, Diagnóstico e Manejo da Fibrilação Atrial em Pacientes com Câncer: Evidências Atuais e Perspectivas Futuras

**DOI:** 10.36660/abc.20201362

**Published:** 2022-07-29

**Authors:** Pedro Gonçalves-Teixeira, Telma Costa, Isabel Fragoso, Diogo Ferreira, Mariana Brandão, Adelino Leite-Moreira, Francisco Sampaio, José Ribeiro, Ricardo Fontes-Carvalho

**Affiliations:** 1 Vila Nova de Gaia Hospital Center Departamento de Cardiologia Gaia Portugal Departamento de Cardiologia, Vila Nova de Gaia Hospital Center, Gaia – Portugal; 2 Universidade do Porto Faculdade de Medicina Departamento de Fisiologia Porto Portugal Departamento de Fisiologia, Faculdade de Medicina, Universidade do Porto, Porto – Portugal; 3 Centro Hospitalar Vila Nova de Gaia Clínica Cardio-Oncológica Gaia Portugal Clínica Cardio-Oncológica, Centro Hospitalar Vila Nova de Gaia, Gaia – Portugal; 4 Centro Hospitalar Vila Nova de Gaia Departamento de Oncologia Gaia Portugal Departamento de Oncologia, Centro Hospitalar Vila Nova de Gaia, Gaia – Portugal; 5 Unidade de Atenção Primária à Saúde Aracetti Arazede Portugal Unidade de Atenção Primária à Saúde Aracetti, Arazede – Portugal; 6 Hospital Universitário São João Departamento de Cirurgia Cardiotorácica Porto Portugal Departamento de Cirurgia Cardiotorácica, Hospital Universitário São João, Porto – Portugal; 7 Universidade do Porto Faculdade de Medicina Unidade de Pesquisa Cardiovascular Porto Portugal Unidade de Pesquisa Cardiovascular (UnIC), Faculdade de Medicina, Universidade do Porto, Porto – Portugal

**Keywords:** Fibrilação Atrial, Câncer, Cardiotoxicidade, Rastreamento, Cardio-Oncologia, Arritmias Cardíacas, Anticoagulantes, Coagulação Sanguínea

## Abstract

A fibrilação atrial (FA) é a arritmia cardíaca sustentada mais comum na população geral, tendo uma alta carga de morbimortalidade, e isso também é válido para pacientes com câncer. A associação entre FA e câncer vai ainda mais longe, com alguns estudos sugerindo que a FA pode ser um marcador de câncer oculto. Há, no entanto, uma notável escassez de dados sobre os desafios específicos do manejo da FA em pacientes com câncer. O reconhecimento e o manejo imediatos da FA nesta população especial podem diminuir a morbidade relacionada à arritmia e ter um importante benefício prognóstico. Esta revisão se concentrará nos desafios atuais de diagnóstico e manejo da FA em pacientes com câncer, com ênfase especial nas estratégias e dispositivos de rastreamento da FA e na terapia de anticoagulação com anticoagulantes orais não antagonistas da vitamina K (NOACs) para prevenção tromboembólica nesses pacientes. Alguns *insights* sobre as perspectivas futuras para a prevenção, diagnóstico e tratamento da FA nesta população especial também serão abordados.

## Introdução

A Cardio-oncologia emergiu como um campo clínico crucial no manejo de pacientes com câncer na última década. As clínicas de cardio-oncologia agora oferecem um atendimento clínico verdadeiramente centrado no paciente e se mostraram úteis na prevenção da toxicidade cardiovascular relacionada à terapia do câncer

Tradicionalmente, as clínicas de oncologia limitavam-se ao conhecimento da potencial toxicidade aos cardiomiócitos e o risco de insuficiência cardíaca subsequente. Agora temos uma visão cada vez mais amadurecida da plêiade de cardiotoxicidade relacionada à terapia do câncer. Isso inclui um amplo espectro de complicações inflamatórias, tromboembólicas e arrítmicas.

### Carga da FA

A fibrilação atrial (FA) é reconhecida como a arritmia cardíaca sustentada mais comum, com prevalência de aproximadamente 0,5 a 2% da população geral. Pacientes com FA têm um risco cinco vezes maior de acidente vascular cerebral (AVC) e um risco três vezes maior de insuficiência cardíaca. Além disso, a FA é um preditor independente de morbidade e mortalidade cardiovascular.^1,2^

Os fatores que predispõem ao desenvolvimento de FA incluem o envelhecimento (a prevalência de FA chega a 10% em pacientes com mais de 80 anos),^3^ distúrbios cardiovasculares como hipertensão, valvopatia, insuficiência cardíaca, hipertensão pulmonar e uma variedade de comorbidades não-cardiovasculares como diabetes, doença pulmonar crônica, apneia obstrutiva do sono, doença renal crônica, disfunção tireoidiana, doença inflamatória intestinal, entre outras.

A associação entre FA e câncer é reconhecida há muito tempo e é de certa forma esperada com base no aumento da prevalência de câncer com o envelhecimento e na alta frequência de comorbidades que predispõem à FA em pacientes com câncer.

Vários estudos de coorte de base populacional mostraram a clara associação bidirecional entre essas entidades. Uma meta-análise recente mostrou que a taxa de diagnóstico de câncer foi três vezes maior nos primeiros três meses após o diagnóstico de FA. Por outro lado, o risco de FA estava particularmente aumentado nos primeiros três meses após o diagnóstico de câncer (OR 7,62, IC 3,08 a 18,88).^4,5^ Além disso, em um grande estudo caso-controle populacional com 28.833 casos de FA, 0,59% deles foram diagnosticados com câncer colorretal nos 90 dias anteriores ao diagnóstico de FA, em comparação com apenas 0,05% dos controles.^6^ Outro estudo de coorte também descobriu que a FA estava associada a uma maior taxa de incidência de diagnóstico de câncer nas duas décadas subsequentes de seguimento e, mais uma vez, isso é particularmente verdadeiro dentro do período de 90 dias após o diagnóstico de FA. Nesse período de 90 dias, os homens apresentaram risco aproximadamente três vezes maior de ter um diagnóstico de câncer, enquanto as mulheres tiveram um risco quatro vezes maior.^7^ Em um estudo observacional publicado recentemente, abrangendo 4.324.545 indivíduos, dos quais 316.040 apresentavam diagnóstico de câncer, a FA permaneceu independentemente associada a todos os principais subtipos de câncer.^8^ A prevalência geral de FA foi de 1,74% entre os pacientes com câncer versus 0,37% na população geral, e essa diferença aumentou com a idade. A força da associação diminuiu ao longo do tempo a partir do diagnóstico de câncer, mas permaneceu significativa mesmo após 5 anos (taxa de incidência de 3,4 do dia 0 ao dia 90 e 1,1 de 2 a 5 anos a partir do diagnóstico de câncer). Outro estudo de coorte nacional concluiu que a FA estava fortemente associada ao câncer metastático.^9^

Sabe-se que a FA pode ser uma condição assintomática, principalmente em idosos. A frequente natureza paroxística da FA complica ainda mais o seu reconhecimento precoce. Estudos demonstraram que até 45% de todos os AVCs relacionados à FA ocorreram em pacientes com FA assintomática e desconhecida.^10^ Acredita-se que o risco significativo de complicações tromboembólicas da FA seja ainda maior em pacientes com câncer, em quem geralmente prevalece um estado pró-coagulante.

O rastreamento e a investigação da FA podem ter um papel potencial na prevenção de complicações se o tratamento adequado for prescrito precocemente.

Por outro lado, como a associação entre FA e câncer vai ainda mais longe, alguns estudos sugerem que a FA pode ser um marcador de câncer oculto. Os autores de uma meta-análise composta por 5 estudos observacionais de base populacional, incluindo mais de 5.500.000 pacientes, recomendaram que pacientes com FA de início recente fossem avaliados para câncer oculto.^5^ Atualmente isso é bastante controverso, e foi refutado por outros autores.^7,11^

Esta revisão se concentrará nos desafios atuais de diagnóstico e manejo de FA em pacientes com câncer, com ênfase especial no rastreamento de FA e terapia de anticoagulação para prevenção de AVC tromboembólico nesses pacientes. Alguns *insights* sobre as perspectivas futuras para a prevenção, identificação e tratamento da FA nesta população especial também serão fornecidos.

### FA e Câncer: ligações fisiopatológicas propostas

**Figura 1:**
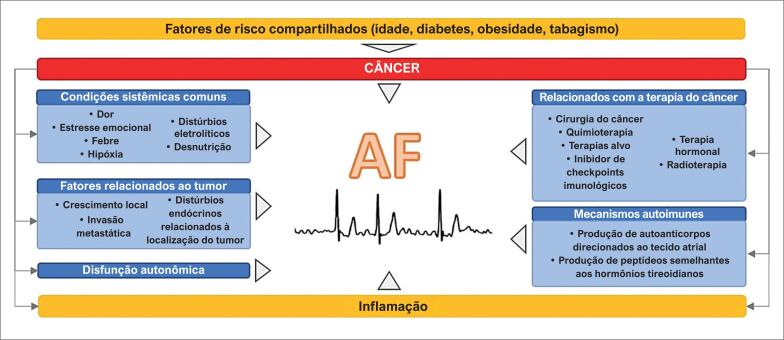
A interação multifatorial e bidirecional entre câncer e fibrilação atrial. Veja o texto para mais detalhes.

Múltiplas relações fisiopatológicas foram propostas para explicar a forte associação entre as duas entidades (Figura 1).

A existência de fatores de risco compartilhados para câncer e FA – como doenças cardiovasculares preexistentes, envelhecimento, obesidade, diabetes, consumo de álcool e tabagismo – pode explicar uma proporção significativa dessa relação epidemiológica.

Além disso, pacientes com câncer frequentemente experimentam dor, hipóxia, anormalidades eletrolíticas e desnutrição, todas as quais podem provocar várias anormalidades autonômicas e endócrino-metabólicas que contribuem para a FA.^12^

No nível dos átrios, o crescimento tumoral primário ou metastático pode provocar compressão ou invasão local, ambos com potencial para desencadear FA.

Tem sido sugerido que o câncer aumenta a incidência de FA através da produção anormal de peptídeos semelhantes aos hormônios tireoidianos.^13^ Uma variedade de síndromes paraneoplásicas pode levar a desarranjos endócrinos ou metabólicos e preparar o terreno para o desenvolvimento de FA. Outros mecanismos autoimunes que têm como alvo o tecido atrial foram postulados.^14^

O câncer oculto não diagnosticado, acompanhado pelo tônus autonômico alterado e um estado pró-inflamatório, pode preceder a FA e explicar, pelo menos em parte, a associação. Em alguns desses casos, a terapia de anticoagulação pode desmascarar o distúrbio neoplásico, promovendo eventos hemorrágicos relacionados ao tumor. Além disso, estando mais expostos a exames médicos e testes diagnósticos, pacientes com câncer recentemente diagnosticados têm maior probabilidade de diagnóstico de FA de início recente.

Há uma grande quantidade de evidências ligando a FA a distúrbios inflamatórios. A alta prevalência de FA no pós-operatório e em estágios agudos de infarto agudo do miocárdio (IAM) ou miocardite, fornecem uma valiosa visão sobre a relação entre FA e inflamação. Estudos histológicos exploraram ainda mais essa questão, com pacientes com FA apresentando infiltrados de células inflamatórias no endocárdio do átrio direito, o que não foi observado nos controles.^15^ Vários estudos avaliaram biomarcadores inflamatórios nesse contexto, mostrando que a proteína C reativa (PCR).^16,17^ interleucina 2 (IL-2),^18^ interleucina 6 (IL-6),^19^ fator de necrose tumoral-α (TNF-α) e proteína quimiotática de monócitos 1 (MCP-1)^20^ estavam significativamente elevados em pacientes com FA quando comparados aos controles. A associação entre câncer e inflamação, sendo notavelmente robusta,^21,22^ permite levantar a hipótese de que a inflamação é provavelmente um substrato comum para FA e câncer em alguns pacientes.^23^

A FA é frequentemente observada após a terapia cirúrgica para câncer, e isso é particularmente evidente após a ressecção pulmonar para câncer de pulmão, com um grande estudo observacional mostrando uma prevalência de 12,6%.^24^ Isso também foi documentado após cirurgia para câncer de esôfago, colorretal e de mama.^25-27^

Finalmente, vários medicamentos anticancerígenos amplamente utilizados têm sido associados a um risco aumentado de FA incidente (Tabela 1). Um interesse renovado neste campo surgiu após os primeiros relatos de FA relacionada ao ibrutinibe, um inibidor de tirosina quinase (TKi) utilizado em pacientes com leucemia linfocítica crônica, linfoma de células do manto e outras malignidades hematológicas. A incidência de FA em pacientes sob terapia com ibrutinibe variou de 3% a 16%.^28^ Os efeitos antiplaquetários do ibrutinibe, que parece inibir as etapas iniciais de adesão e ativação plaquetárias,^29^ podem representar desafios terapêuticos quando é necessário tomar uma decisão sobre a anticoagulação. Tem sido sugerido que a terapia de privação androgênica utilizada para tratar o câncer de próstata pode levar a uma maior incidência de FA, possivelmente relacionada ao hipogonadismo relacionado à hormonoterapia.^30^ Esse risco foi mais pronunciado com a abiraterona, medicamento que também bloqueia as enzimas CYP17, podendo causar hipermineralocortisolismo, promovendo hipocalemia e FA.^31^ Mais recentemente, os inibidores do checkpoint imunológico (ICI, *immune checkpoint inhibitors*), também foram associados à FA de início recente devido à sua propensão a causar inflamação miocárdica e pericárdica através de mecanismos autoimunes.^32^ Outros efeitos colaterais autoimunes dos ICIs, como tireoidite, também podem predispor ao desenvolvimento de FA.

**Tabela 1:** Frequência relatada de fibrilação atrial induzida por terapia de câncer

Classe terapêutica	Agente medicamentoso	Frequência de FA relatada
Agentes alquilantes	Antraciclinas	0,55 – 10,3%
	Melfalano	10,8 – 33%
	Bussulfan	6,4%
	Ciclofosfamida	2%
Antimetabólitos	5-Fluorouracil	5%
	Capecitabina	0,5 – 1,1%
	Gemcitabina	0-8,1% (*)
Taxanes	Paclitaxel	0,18 - 1%
Imunomoduladores	Talidomida	4,7%
	Lenalidomida	4,6 - 7%
Derivados de platina	Cisplatina	10-32%
Inibidores da tirosina quinase	Ibrutinibe (BTK)	3-16%
	Nilotinibe (BCR-ABL1)	0,8%
	Ponatinibe (BCR-ABL1)	3-7%
	Vemurafenibe (BRAF)	1,5%
	Imatinibe (BCR-ABL1)	0,55 – 33%
	Dasatinibe (BCR-ABL1)	5,6%
	Sorafenibe (VEGFR)	5,1% (**)
Inibidores do proteassoma	Bortezomibe	2,2%
	Carfilzomibe	3,2 – 3,8%
Anticorpos monoclonais	Trastuzumabe (HER2/ERBB2)	1,2%
	Bevacizumabe (VEFG)	2,2%
	Cetuximabe (EGFR/HER1)	4,8%
	Alentuzumabe (CD52)	1,2%
	Rituximabe (CD20)	1%
Outros	Interleucina 2	4,3 – 8%
ICIs	Nivolumabe (anti-PD1)	13%
	Pembrolizumabe (anti-PD1)	
	Ipilimumabe (anti-CTLA4)	
Terapia com células CAR-T		2,2%
Hormonoterapia	Degarelix	2%
	Abiraterona	1 – 5%
Radioterapia		0,5 –3,2%

(*) Incidência de FA de 0% quando usada isoladamente, 8% quando associada à vinorelbina.

(**) A prevalência relatada foi encontrada em associação com 5-FU, em estudo de fase II. É interessante lembrar que essa associação não é utilizada atualmente na prática clínica diária.

A radioterapia torácica está associada à fibrose miocárdica potencialmente causando uma cardiomiopatia restritiva em longo prazo, e a associada elevação da pressão de enchimento favorece o desenvolvimento de FA. O aumento da fibrose miocárdica a nível dos átrios pode preparar o terreno para a subsequente remodelação mecânica e/ou elétrica, eventualmente causando a FA.

Deve-se reconhecer, no entanto, que a incidência real de FA relacionada à terapia do câncer provavelmente será subestimada, pois o monitoramento de rotina é raramente realizado ou compreende apenas um ECG de 12 derivações de registro único.

### A justificativa para o rastreamento da FA

A FA não raramente é uma condição assintomática, e o risco de AVC ou morte foi semelhante entre a FA sintomática e a FA silenciosa.^33,34^ Até 5% dos indivíduos com FA têm um AVC como manifestação clínica inicial de sua arritmia.^35^ Isso pode representar cerca de um terço de todos os AVCs relacionados à FA. A FA está associada ao aumento do risco de mortalidade na população geral,^36-39^ e isso também se mostrou verdadeiro em pacientes com câncer.^40,41^

A prevenção de AVC tromboembólico devido à introdução precoce de anticoagulante oral em pacientes de risco é talvez o benefício mais plausível dos programas de rastreamento de FA.^42^ Outros benefícios teóricos propostos do reconhecimento e manejo precoce da FA incluem redução da morbidade e hospitalizações relacionadas à FA e redução da mortalidade relacionada à FA.

O valor agregado do rastreamento oportunista/sistemático versus o padrão de cuidado para detectar FA silenciosa na população geral está bem estabelecido, e as taxas de FA recém-diagnosticada variaram de 0,5 a 3,9% na maioria dos estudos.^43-49^ O ganho crescente dos programas de rastreio parece estar mais intimamente relacionado com a população rastreada e a duração do rastreamento do que com as características dos dispositivos/testes específicos.

Fatores como idade,^44^ histórico anterior de AVC tromboembólico,^50,51^ escore CHA2DS2-VASc^52,53^ e níveis de NT-proBNP,^54,55^ têm sido propostos como potencialmente úteis para otimizar o “número necessário para rastreamento” desses programas, possivelmente permitindo a melhora do benefício clínico líquido e custo-benefício.

Curiosamente, o escore CHA2DS2-VASc não apenas prediz o risco de AVC entre pacientes com FA conhecida, mas também tem um desempenho bastante razoável na previsão de FA recém-diagnosticada. Isso pode ser útil como uma porta de entrada para programas de rastreamento, não apenas (1) ajudando a selecionar os pacientes com maior probabilidade pré-teste de FA silenciosa, mas também porque (2) garante que todos os casos detectados obtenham benefício clínico da prescrição do anticoagulante oral (ACO).

O ensaio clínico STROKESTOP incluiu indivíduos de 75 e 76 anos, selecionando participantes com um escore CHA2DS2-VASc de pelo menos 2 pontos (idade>75). FA previamente desconhecida foi encontrada em 0,5% da população rastreada em seu primeiro ECG, enquanto registros de ECG intermitentes aumentaram a detecção de FA em 4 vezes.^44^

O estudo STROKESTOP II acrescentou a isso o uso do NT-proBNP, em uma estratégia gradual para rastreamento de FA em indivíduos de 75 e 76 anos. O grupo de alto risco (NT-proBNP ≥125 ng/L) recebeu um rastreio de ECG estendido, enquanto o grupo de baixo risco realizou apenas um registro de ECG. No grupo de alto risco, 4,4% tiveram FA recém-diagnosticada.^56^

Mesmo em coortes com maior risco de AVC tromboembólico (ou seja, aqueles com AVC embólico anterior de origem indeterminada), o tratamento empírico com ACO não demonstrou redução no AVC recorrente. Isso reforça a importância do registro eficaz da FA antes da implementação de tais terapias,^57,58^ mesmo em coortes de alta prevalência e alto risco, como pacientes com câncer. Em pacientes com FA documentada, a terapia com ACO reduziu as taxas de AVC em dois terços.^50^

### Estratégias para o rastreamento de FA

**Figura 2:**
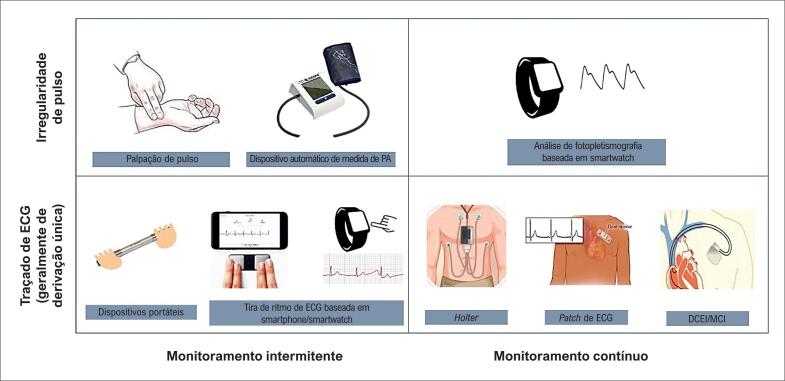
Vários métodos estão disponíveis para rastreamento ambulatorial de fibrilação atrial. PA: pressão arterial. DCEI: dispositivo cardíaco eletrônico implantável. MCI: monitor cardíaco implantável.

Vários métodos estão disponíveis para rastreamento de FA (Figura 2). O método mais simples de rastreamento para FA é a tomada de pulso, que fornece boa sensibilidade, mas uma especificidade modesta (intervalo relatado de 65 a 91%). Outras abordagens incluem dispositivos automatizados de pressão arterial (aqueles capazes de realizar análises oscilométricas),^59^ dispositivos não invasivos para registro de ECG de derivação única e *patches* de monitoramento do ritmo cardíaco.

Mais recentemente, o monitoramento ambulatorial baseado em smartphone e smartwatch introduziu a capacidade de monitoramento ativado pelo paciente sem a necessidade de dispositivos vestíveis e por períodos indefinidos. O smartwatch mostrou resultados promissores em um estudo com 419.000 participantes, sobre rastreamento em massa para FA. Padrões de ritmo irregulares foram detectados em 0,52% dos participantes, e isso levou à confirmação posterior com um eletrocardiograma (ECG) por *patch*. O valor preditivo positivo dos ritmos irregulares detectados pelo smartwatch como possível FA foi de 0,71. Deve-se notar, no entanto, o perfil etário desfavorável dos indivíduos inscritos, em sua maioria jovens (52% com menos de 40 anos e apenas 6% com 65 anos ou mais).^46^

A análise de ritmo baseada em inteligência artificial é frequentemente dependente de algoritmos heterogêneos e, portanto, é necessária a validação subsequente dos achados. Isso se aplica não apenas à análise de pletismografia para irregularidades de onda de pulso, mas também para geração de ECG de derivação única de alguns dispositivos, cuja precisão diagnóstica ainda não substitui o julgamento humano. Isso pode representar um desafio para os sistemas de saúde, já frequentemente confrontados com escassez de recursos humanos, uma vez que a grande quantidade de dados gerados por esses dispositivos acaba por necessitar de validação.

Até o momento, estudos randomizados de rastreamento de FA não demonstraram redução no AVC ou outros desfechos importantes. Deve-se reconhecer, no entanto, que nenhum desses estudos tinha poder adequado para demonstrar tal efeito. Vários estudos estão atualmente em andamento, buscando fornecer insights sobre esse importante tópico (SAFER,^60^ DANCANVAS,^61^ LOOP,^62^ GUARD-AF^63^).

Duas importantes desvantagens têm sido apontadas a respeito das estratégias de rastreamento da FA. A primeira diz respeito ao risco de resultados falso-positivos e potencial de risco aumentado de sangramento em pacientes nos quais a ACO não traz benefício clínico. As consequências psicológicas esperadas de um resultado falso positivo, no que diz respeito aos níveis de ansiedade e diminuição da qualidade de vida, podem ter importância redobrada em pacientes oncológicos. A segunda enfatiza o significado clínico incerto de episódios curtos de FA documentados com modalidades de rastreamento prolongado. De fato, esses episódios de arritmia de curta duração podem não representar risco aumentado de eventos tromboembólicos.^64^

Após a detecção de FA de início recente com qualquer estratégia de rastreamento, deve-se ressaltar, no entanto, que a confirmação de FA por ECG ainda é obrigatória nas diretrizes.^2^

### Recomendações atuais para rastreamento de FA

A Sociedade Europeia de Cardiologia (ESC, *European Society of Cardiology*) recomenda o rastreamento oportuno da FA através da tomada de pulso ou fita de ECG em pacientes com >65 anos, com Classe de Recomendação (COR, *Class of Recommendation*) I e Nível de Evidência (LOE, *Level of Evidence*) B.^2^ De acordo com as mesmas recomendações, o rastreamento sistemático por ECG pode ser considerado para detectar FA em pacientes com 75 anos ou mais ou naqueles com alto risco de AVC (COR IIb, LOE B).Um documento de consenso da *European Heart Rhythm Association* (EHRA) acrescenta que o rastreamento para FA é recomendado em populações de alto risco, devido ao seu custo-efetividade.^42^

Em contrapartida, a *United States Preventive Services Task Force* (Força-Tarefa de Serviços Preventivos) dos Estados Unidos afirma que as evidências atuais são insuficientes para avaliar o equilíbrio entre benefícios e danos do rastreamento de FA com eletrocardiografia.^65^

Apesar da alta carga de FA em pacientes com câncer, não há recomendações específicas sobre o rastreamento de FA nesses pacientes.

### Rastreamento de FA em pacientes com câncer: quais são as evidências?

Há uma surpreendente escassez de dados sobre o rastreamento de FA em pacientes com câncer. Além disso, malignidade atual e/ou exposição à quimioterapia ou radioterapia foram critérios de exclusão em alguns estudos sobre rastreamento de FA.^62,66,67^

Curiosamente, a maioria dos estudos de rastreamento de FA nem sequer relata a prevalência de câncer quando se trata de caracterização da população rastreada. Entre os poucos estudos que relatam a prevalência de câncer no início do estudo, não existe uma descrição clara sobre a taxa de FA recém identificada e/ou o “número necessário rastrear” nesses pacientes.

Um estudo transversal nacional da Irlanda rastreou aleatoriamente 2.200 pacientes com 70 anos ou mais com um monitor de ECG de 3 derivações na atenção primária. A taxa de incidência de FA recém-diagnosticada foi de 1,2%. Este estudo relatou uma prevalência de câncer de pulmão de 0,3% na população geral rastreada, mas, uma vez mais, não há dados disponíveis sobre a taxa de incidência de FA recém-identificada para esses pacientes.

### Manejo da FA em pacientes com câncer

Os princípios gerais de prevenção e tratamento da FA e recomendações gerais de manejo também se aplicam a pacientes com câncer. Por uma questão de consistência, o autor seguirá a abordagem “ABC” recomendada pelas diretrizes para o tratamento da FA (A: evitar acidente vascular cerebral, anticoagulação (*avoid stroke, anticoagulation*); B: melhor controle dos sintomas, incluindo decisões compartilhadas pelo paciente sobre estratégias de controle de frequência ou ritmo (*better symptom management, including patient-shared decisions on rate or rhythm control strategies*); C: redução do risco cardiovascular e de comorbidade (*cardiovascular and comorbidity risk reduction*). Também abordamos algumas particularidades dos pacientes com câncer que merecem consideração.

#### Regime antitrombótico

Em pacientes com FA da população geral, o risco de AVC isquêmico é estratificado com precisão satisfatória pelo escore CHA2DS2-VASc, e pacientes com escore ≥1 (exceto para o sexo feminino isolado), são considerados como tendo risco/benefício favorável sob terapia com ACO.^2^ Isso deve ser equilibrado com o risco de sangramento em cada paciente. O escore HAS-BLED foi proposto para avaliação do risco de sangramento na população geral.^68^ A escala de avaliação de risco HEMORR2HAGES tem a característica única de incluir o câncer como fator de risco para sangramento na FA, mas carece de validação externa. A modificação do fator de risco é de extrema importância para minimizar o risco de sangramento. Além do desempenho abaixo do ideal e da capacidade discriminatória, os inúmeros escores de risco de sangramento disponíveis têm o mérito de destacar esses fatores de risco modificáveis.

A terapia com ACO reduz o risco de AVC isquêmico em cerca de 60%. Vários estudos clínicos de referência evidenciaram o perfil de segurança superior de NOACs *versus* AVKs (antagonistas da vitamina K) com eficácia comparável na população geral.^69-72^ Entretanto, esses estudos, direta (excluindo pacientes submetidos a quimioterapia/radioterapia ativa) ou indiretamente (não permitindo a inclusão de indivíduos com sobrevida esperada de <12 meses), excluíram pacientes com câncer ativo.

**Figura 3:**
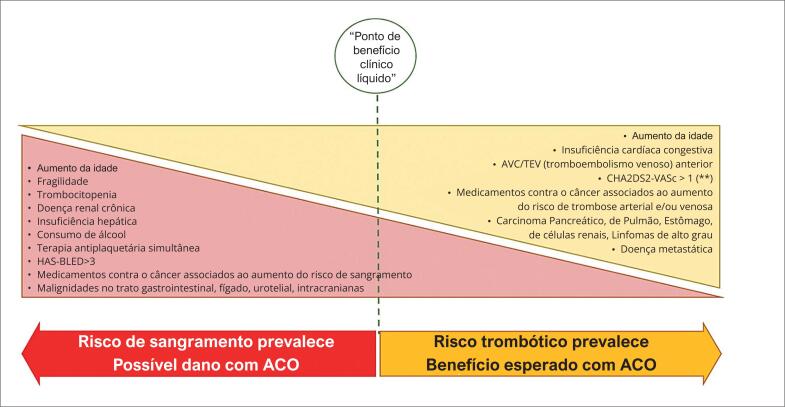
Pacientes com câncer e FA apresentam risco trombótico e hemorrágico simultaneamente alto. Os fatores dos pacientes, bem como os riscos específicos do tumor e os efeitos adversos da terapêutica do câncer, representam desafios adicionais. A indicação de anticoagulação para esses pacientes deve ser individualizada, e diversos fatores, não incluídos nos escores de risco clássicos, devem ser considerados. (*) Alcaloides vinca, agentes alquilantes, anticorpos monoclonais (aflibercepte, bevacizumabe, ramucirumabe, trastuzumabe emtansina), antiestrogênicos, antimetabólitos (pentostatina), antraciclinas, bleomicina, camptotecinas, carfilzomibe, epipodofilotoxinas, ibrutinibe, BCR-ABL, BRAF e inibidores de VEGF/VEGFR, interleucinas, L-asparaginase, ruxolitinibe, taxanos, temozolomida, ciclofosfamida, ifosfamida, megestrol, tamoxifeno. (**) O escore CHA2DS2-VASc é um forte preditor de eventos tromboembólicos em pacientes com FA previamente conhecida, mas com desempenho insatisfatório para a previsão de risco de AVC em pacientes com câncer e FA recém-diagnosticada. Consulte o texto para obter mais detalhes. (***) Agentes alquilantes (carboplatina, ciclofosfamida, cisplatina, estramustina, oxaliplatina, temozolomida), análogos do hormônio liberador de gonadotropina, antiandrogênicos, anticorpos monoclonais (aflibercepte, bevacizumabe, cetuximabe, panitumumabe), antraciclinas, antimetabólitos (capecitabina, 5-fluorouracil, gencitabina, metotrexato, pentostatina), imunomoduladores (lenalidomida, pomalidomida, talidomida), inibidores de aromatase, bleomicina, inibidores de proteína quinase (axitinibe, lenvatinibe, pazopanibe, sorafenibe, sunitinibe), inibidores de mTOR, inibidores de proteassomo (carfilzomibe), irinotecano, taxanos, tasonermina, tretinoína, megestrol, progestagênios, raloxifeno, tamoxifeno, vinflunina, vorinostat, agentes estimuladores da eritropoiese e fatores estimulantes de colônias de granulócitos.

Os eventos trombóticos são a segunda causa de mortalidade em pacientes com câncer.^73^ No entanto, o câncer e muitas de suas características de risco trombótico não são incorporados no cálculo do escore CHA2DS2-VASc. Além disso, o risco de sangramento associado ao câncer pode, teoricamente, mudar o “ponto de benefício clínico líquido” da ACO nesses pacientes em direção a um escore CHA2DS2-VASc mais alto (Figura 3).

Análises conflitantes têm sido feitas em relação ao desempenho dos escores CHADS2 e CHA2DS2-VASc em pacientes com câncer e FA. Em um estudo que incluiu mais de 120.000 pacientes, aqueles com câncer e um baixo escore CHA2DS2-VASc (0-1) tiveram um risco maior de AVC do que pacientes sem câncer, mas naqueles com escore ≥2, o risco de AVC foi semelhante entre pacientes com e sem câncer.^74^

Em um estudo com cerca de 2.000 pacientes, o escore CHADS2 foi mais preditivo de risco aumentado de AVC em pacientes com câncer e FA pré-existente (cada aumento de um ponto foi associado a um risco quase 40% maior de AVC) do que o escore CHA2DS2-VASc.^75^ No mesmo estudo, entretanto, ambos os escores previram com precisão o risco de acidente vascular cerebral e a sobrevida. Curiosamente, em outro estudo,^76^ o escore CHADS2 não apresentou poder para prever tromboembolismo em pacientes com câncer com FA de início recente.

Por outro lado, pacientes com câncer recente apresentaram maior risco de sangramento, independente do escore CHA2DS2-VASc.^74^ Pacientes com câncer têm um risco visivelmente maior de eventos hemorrágicos, pela localização da malignidade, cirurgia do câncer, trombocitopenia, disfunção plaquetária, agentes quimioterápicos, radioterapia, insuficiência renal ou hepática iatrogênica e/ou relacionada ao tumor, supressão da medula óssea (pelo distúrbio neoplásico ou terapêutica relacionada ao câncer), coagulação intravascular disseminada ou hiperfibrinólise em subconjuntos específicos, mucosite e doença de von Willebrand adquirida. No registro de Riete,^77^ sangramento prévio, clearance de creatinina <;30 mL/min, imobilidade ≥4 dias e doença metastática foram os preditores mais importantes de sangramento maior em pacientes com câncer submetidos à terapia de anticoagulação.

Em uma grande análise de dados de registro, pacientes com câncer tiveram um aumento de duas a seis vezes no risco de sangramento em comparação com pacientes sem câncer.^78^ A taxa de AVC isquêmico foi, no entanto, comparável.

Evidências de ensaios clínicos randomizados comparando NOACs com antagonistas da vitamina K (AVK) ou heparina de baixo peso molecular (HBPM) para prevenção tromboembólica em pacientes com câncer e FA não estão disponíveis até o momento.

Recentemente vários ensaios clínicos randomizados (ECRs) enfatizaram o perfil de eficácia e segurança do NOAC para profilaxia de tromboembolismo venoso^79,80^ e tratamento^81-83^ em pacientes com câncer, em comparação com heparinas de baixo peso molecular (HBPM). Em todos esses estudos, o risco de sangramento minor foi maior com NOAC *versus* HBPM (causado pela maior taxa de sangramento gastrointestinal). O risco de sangramento major foi semelhante entre as duas classes de medicamentos em alguns estudos (Caravaggio^83^ e SELECT-D^82^), mas um risco aumentado com o uso de NOAC foi observado em um estudo (Hokusai VTE Cancer^81^). Até certo ponto, uma extrapolação cautelosa pode ser feita a partir desses estudos, mas a fisiopatologia tromboembólica única em pacientes com FA merece estudos dedicados.

Dados observacionais recentes de uma coorte de 16.096 pacientes com FA e câncer sugerem que os NOACs podem ser pelo menos tão eficazes quanto a varfarina na prevenção de AVC isquêmico e ter um perfil de sangramento mais seguro.^84^

Um resumo de várias sub-análises dos principais estudos clínicos de terapia de FA com ACO em pacientes com câncer é apresentado na tabela 2. Em uma sub-análise do estudo ARISTOTLE, a segurança e eficácia de apixabana *versus* varfarina foram comparáveis entre pacientes com e sem câncer ativo.^85^ Curiosamente, os pacientes com câncer obtiveram um benefício maior da terapia com apixabana para o *endpoint* composto de AVC/embolismo sistêmico, infarto do miocárdio (IM) e morte. Esses resultados foram replicados em uma análise de 1.153 pacientes inicialmente incluídos no estudo ENGAGE AF-TIMI 48, que desenvolveu neoplasias novas ou recorrentes em um seguimento médio de 495 dias.^86^ No geral, o perfil de eficácia e segurança do edoxaban em relação à varfarina foi preservado.

**Tabela 2:** NOACs versus varfarina para prevenção de acidente vascular cerebral em pacientes com fibrilação atrial

NOAC	*Endpoint* de eficácia primária vs. Varfarina RR [IC 95%]		*Endpoint* de segurança primário* vs. Varfarina RR [IC 95%]	
	População Geral **	Câncer ***	População Geral **	*Câncer* ***
Dabigatrana	0,91 [0,53-0,82] †	0,14 [0,03 - 0,57] §	0,93 [0,81-1,07] †	0,23 [0,07-0,74] §
Rivaroxabana	0,79 [0,66-0,96]	0,52 [0,22-1,21]	1,03 [0,96-1,11]	1,09 [0,82-1,44] ††
Apixabana	0,79 [0,66-0,95]	1,09 [0,56-2,26]	0,69 [0,60-0,80]	0,80 [0,56-1,14] ††
Edoxabana	0,79 [0,63-0,99] ‡	0,60 [0,31-1,15] ‡	0,87 [0,73-1,04] ‡	0,98 [0,69–1,40] ‡

* Resultados de sangramento maior, a menos que especificado de outra forma.

** Dados de ECRs de referência.

*** Dados de sub-análise post-hoc ou estudos observacionais.

§ Resultados de um estudo retrospectivo observacional, que incluiu 140 pacientes em uso de dabigatrana, e observou dois acidentes vasculares cerebrais isquêmicos e três eventos hemorrágicos maiores neste braço de estudo (Kim K, et al. 2018].

† São apresentados os resultados para a dosagem de 150mg de Dabigatrana.

†† Sangramento grave ou não grave clinicamente relevante.

‡ São apresentados os resultados para a dosagem de 60mg de Edoxabana.

Em uma meta-análise recentemente publicada que incluiu mais de 20.000 pacientes com FA e câncer recebendo ACO, os NOACs apresentaram taxas mais baixas ou semelhantes de eventos tromboembólicos e hemorrágicos em comparação com a varfarina (37% de redução do risco de AVC, 27% de redução do risco de sangramento maior).^87^ Esses resultados ainda são exploratórios e devem ser interpretados com cautela até que os ECRs estejam disponíveis. Uma limitação importante diz respeito aos dados limitados sobre o estadiamento do câncer, o que pode ter levado a fatores de confusão não controlados se o tipo de ACO (NOACs *vs*. AVK) variasse de acordo com o estadiamento do câncer. Além disso, pacientes com maior gravidade da doença (ou seja, aqueles com expectativa de vida reduzida) foram indiretamente excluídos pela análise, pois foram excluídos pelos numerosos estudos incluídos.

A avaliação individualizada do perfil de risco trombótico e de sangramento, comorbidades e interações medicamentosas esperadas em cada paciente continua sendo fundamental, seja antes do início da estratégia com ACO, ao avaliar a necessidade de ajuste de dose ou modificação do esquema, ou até mesmo a descontinuação da terapia.

Equilibrar o risco trombótico e/ou de sangramento permanece particularmente desafiador em cenários específicos, de acordo com as comorbidades, localização do tumor, estadiamento e terapias relacionadas ao câncer, alguns das quais são abordados na Figura 3. Embora, no momento, não existam dados para orientar a escolha de anticoagulantes específicos na maioria desses cenários extremos, parece aconselhável abster-se de usar rivaroxabana, dabigatrana ou edoxabana em pacientes com câncer gastrointestinal com alto risco de sangramento.

### Encerramento do AAE

O encerrramento percutâneo do apêndice atrial esquerdo (AAE) não foi inferior à varfarina para a prevenção de eventos tromboembólicos, e pode ser considerado naqueles pacientes com maior risco de AVC que apresentam contraindicação para anticoagulação.^88^ A ACO não é nem mesmo necessária no pós-procedimento, pois a terapia antiplaquetária dupla nos primeiros seis meses mostrou-se igualmente segura.^89^ É interessante lembrar que pacientes com trombocitopenia (contagem de plaquetas <100.000) ou anemia (hemoglobina <10g/dL) foram excluídos dos principais estudos que validaram seu uso.

### Especificidades do controle da frequência e do ritmo cardíacos em pacientes com câncer

Para controle sintomático, uma estratégia de controle da frequência cardíaca (controle do batimento) ou restauração e manutenção do ritmo sinusal (controle do ritmo) pode ser razoável. A idade e o estado funcional do paciente, as comorbidades, a duração da FA e as interações medicamentosas previstas com o uso de medicamentos antiarrítmicos e de controle de frequência são aspectos valiosos ao decidir entre as duas estratégias.

A FA de início recente pode surgir no contexto de distúrbios sistêmicos, infecciosos, metabólicos e/ou endócrinos, e sua correção pode ser suficiente para restaurar o ritmo sinusal.

Além desses cenários, na FA hemodinamicamente estável com duração >48h, uma estratégia de controle de frequência cardíaca costuma ser a primeira abordagem. Evidências de ECR de referência que mostram ausência de benefício com uma estratégia de controle de ritmo e um potencial menor de interações medicamentosas com drogas de controle de frequência, foram recentemente questionadas.^90,91^ Aconselha-se uma estratégia moderada de controle da frequência, com um objetivo de frequência cardíaca em repouso de 100-110bpm.^92^ Para este propósito, bloqueadores dos canais de cálcio não-diidropiridínicos (diltiazem, verapamil) e a digoxina apresentam o maior risco de interações medicamentosas relevantes com tratamentos de câncer, e betabloqueadores não significativamente metabolizados pelas enzimas hepáticas (atenolol, nadolol) podem ser preferidos.

Os agentes antiarrítmicos têm um perfil de segurança estreito e, ao escolher um antiarrítmico, deve ser dada atenção às interações graves com medicamentos contra o câncer. Mesmo em pacientes submetidos à cardioversão elétrica planejada para esse fim, os antiarrítmicos podem aumentar a probabilidade de manutenção do ritmo sinusal. A amiodarona é um substrato importante da enzima CYP3A e também um inibidor da glicoproteína P, e deve ser usada com cautela quando estritamente necessário. Antiarrítmicos alternativos em pacientes sem doença estrutural cardíaca (SHD, *structural heart disease*) são o sotalol, a flecainida e a propafenona. A mexiletina (antiarrítmico classe Ib) pode ser considerada em pacientes com SHD.

Dados do registro ORBIT-AF mostram prevalência de 4% de procedimento anterior de ablação por cateter em pacientes com FA com histórico de câncer.^78^ Não há informações se esses procedimentos ocorreram antes ou após o diagnóstico do câncer. Pacientes com histórico de câncer apresentaram menor probabilidade de terem sido submetidos à ablação por cateter de FA, em comparação com aqueles sem histórico de câncer.

O procedimento tem bons resultados em longo prazo em mãos experientes, com baixas taxas de complicações. Pacientes com câncer com expectativa de vida percebida acima de 12 meses seriam teoricamente candidatos plausíveis, visando benefício sintomático e/ou prognóstico.

### Interações medicamentosas

Apesar de serem esperadas menos interações alimentares e medicamentosas com o uso de NOACs em comparação com a varfarina, algumas considerações farmacocinéticas têm relevância clínica. Um transportador intestinal, a glicoproteína P (P-gp), é responsável pela re-secreção gastrointestinal de todos os NOACs. A P-gp também está envolvida na secreção renal de NOACs. Previsivelmente, fortes inibidores da P-gp resultam em níveis plasmáticos aumentados de NOAC.

As vias enzimáticas do citocromo P450 3A4 (CYP3A4) são uma etapa crítica na clearance hepática de rivaroxabana e apixabana. Fortes inibidores da CYP3A4 irão potencialmente aumentar os níveis plasmáticos desses medicamentos.

Como regra geral, inibidores fortes de P-gp e CYP3A4 não são recomendados em combinação com NOACs. Por outro lado, fortes indutores de P-gp e CY3A4, resultando em baixos níveis plasmáticos de NOAC, podem comprometer a eficácia do tratamento. Interações medicamentosas detalhadas e combinações perigosas são detalhadas em outros lugares.^93,94^

Quando evitar a interação medicamentosa grave compromete a eficácia da terapêutica antineoplásica, as heparinas de baixo peso molecular (HBPMs) podem ser consideradas como uma alternativa.

As considerações farmacodinâmicas incluem não apenas o aumento do risco hemorrágico com terapia antiplaquetária simultânea (por exemplo, em pacientes com síndromes coronarianas agudas), mas também o tratamento concomitante com agentes quimioterápicos com atividade antitrombótica. Aconselha-se a avaliação individual do risco trombótico e hemorrágico.

### Ajustes das doses renal e hepática

Em geral, o uso de NOAC não é recomendado na doença renal crônica (DRC) estágio V (*clearance* de creatinina <15mL/min/m^2^). A apixabana é considerada uma alternativa razoável à varfarina nesses pacientes, de acordo com algumas recomendações,^1,95^ mas as evidências de suporte ainda são fracas. Pacientes com DRC estágio IV (CrCl entre 15 e 30 mL/min/m^2^) podem ser tratados com regime de dose reduzida de rivaroxabana, apixabana ou edoxabana. A DRC em estágio III (CrCl 30-60mL/min/m^2^) geralmente exige o ajuste da dose de NOAC, juntamente com as características do paciente que afetam a farmacocinética do fármaco (por exemplo, idade e peso).

Todos os NOACs permanecem contraindicados na doença hepática terminal (cirrose Child-Turcotte-Pugh C) devido à falta de dados. A rivaroxabana também deve ser evitada em pacientes com cirrose hepática Child B.^93^

### Trombocitopenia

Pacientes com câncer com trombocitopenia têm risco aumentado de sangramento, permanecendo em risco aumentado para complicações trombóticas. Até o momento, não surgiram dados robustos sobre qual estratégia de anticoagulação deve ser seguida nesse cenário desafiador. Foi proposta uma estratégia de transfusão de plaquetas ou um regime de anticoagulação com dose modificada com HBPM em pacientes com trombocitopenia grave (contagem de plaquetas <50 x 109/L).^94,96^ Algumas causas de trombocitopenia envolvendo mecanismos imunomediados são caracterizadas por um risco trombótico proeminente, bem como risco hemorrágico. Dito isto, não há consenso sobre um limite inferior de contagem de plaquetas quando se considera a anticoagulação, pois isso é ditado pelo cenário clínico e pelo risco prevalente.

### Modificação do fator de risco

A modificação do fator de risco é crucial na prevenção da FA e na prevenção da recorrência. Isso inclui perda de peso, tratamento do diabetes, controle da hipertensão arterial, reconhecimento e tratamento da apneia do sono, correção da disfunção da tireoide, cessação do tabagismo, evitar o consumo de álcool e tratamento de qualquer doença cardíaca estrutural/isquêmica subjacente.

### Direções futuras

#### Prevenção de FA

Várias intervenções, com foco no estilo de vida e modificação de fatores de risco, levaram a uma redução significativa na carga de FA na população em geral. Essas incluem a perda de peso em pacientes obesos, controle glicêmico ideal em pacientes com DM, manejo da hipertensão e dislipidemia, reconhecimento e tratamento da apneia obstrutiva do sono, cessação do tabagismo e redução do consumo de álcool.^97^ A extensão à qual os pacientes com câncer obtêm o mesmo benefício com essas intervenções ainda não foi determinada, mas a alta carga de fatores de risco cardiovascular clássicos nessa população é um argumento a favor dessas intervenções. O treinamento aeróbico moderado é seguro e proporciona benefícios cardiovasculares e de qualidade de vida (QV) em pacientes com cânce.^98^ Aqueles que integram programas de reabilitação em Cardio-Oncologia experimentam menos eventos adversos relacionados à terapêutica do câncer.^99^

#### Diagnóstico de FA

Algoritmos baseados em inteligência artificial para identificação de alterações sutis de ECG associados ao desenvolvimento futuro de FA (por exemplo, aumento do AE, Síndrome de Bayés)^100^ podem ser úteis na identificação de pacientes que mais podem se beneficiar do rastreamento de FA. O mesmo vale para os parâmetros ecocardiográficos das dimensões e *strain* do AE^101^ e/ou função sistólica e diastólica do VE.^102^ A ressonância magnética cardíaca, permitindo a caracterização morfofuncional atrial, também pode se tornar uma ferramenta crucial no reconhecimento precoce da “miocardiopatia atrial fibrótica”, que está associada à FA incidente e recorrente.^103^ Estudos de associação genômica ampla (GWAS, *Genome-wide association studies*) encontraram várias variantes de genes estruturais atriais associados ao desenvolvimento de FA.^104^ Além disso, as ciências ômicas podem ajudar a refinar nosso conhecimento sobre os processos biológicos subjacentes à FA incidente, talvez ajudando os médicos em sua identificação e tratamento precoces.

Existem modelos de estratificação de risco para toxicidade miocárdica e desenvolvimento de insuficiência cardíaca manifesta, de acordo com as classes de quimioterápicos.^105^ A FA de início recente pode ser objeto de tais ferramentas de estratificação de risco de linha de base, no futuro. Isso pode ajudar os médicos a identificar melhor os pacientes que mais podem se beneficiar do rastreamento de FA.

A eficácia do rastreamento de FA em pacientes com câncer, no que diz respeito à prevenção de eventos cardiovasculares e cerebrovasculares adversos maiores, deve ser abordada em estudos prospectivos com poder adequado. A crescente disponibilidade de dispositivos e aplicativos fáceis de usar, com potencial para rastreamento de longo prazo em um grande número de pacientes, pode impulsionar esse campo de pesquisa.

#### Manejo de FA

Se a ablação da FA traz um benefício prognóstico semelhante em pacientes com câncer com insuficiência cardíaca com fração de ejeção reduzida (ICFEr), como foi demonstrado na população em geral, ainda é desconhecido. Evidências de ensaios clínicos randomizados sobre o uso de NOAC para prevenção de acidente vascular cerebral em pacientes com câncer e FA (em comparação com AVK ou HBPM) também são uma lacuna importante a ser preenchida nos próximos anos.

## Conclusão

As clínicas de cardio-oncologia permitiram que muitas cardiotoxicidades relacionadas à terapêutica do câncer fossem evitadas, reconhecidas precocemente e manejadas de maneira otimizada.

Apesar da alta frequência de FA em pacientes com malignidade ativa, essa condição continua sendo uma comorbidade pouco reconhecida nesses pacientes. Sua natureza paroxística frequente, juntamente com programas de rastreamento deficientes, podem perpetuar essa situação.

O rastreamento da FA em pacientes com câncer pode ter um papel na identificação precoce da FA e na prevenção de eventos tromboembólicos, através da prescrição apropriada de terapia anticoagulante nos indivíduos em risco. A melhor estratégia de rastreamento e o dispositivo ideal para melhorar o resultado desses programas de rastreio ainda não foram estabelecidos.

No futuro, parâmetros clínicos, genéticos, analíticos, eletrocardiográficos e ecocardiográficos podem ajudar a estratificar o risco de desenvolvimento subsequente de FA, auxiliando na seleção de pacientes que merecem protocolos de rastreamento mais rígidos.

Esses pacientes desafiadores, simultaneamente com maior risco trombótico e hemorrágico, merecem estudos clínicos dedicados. O impacto prognóstico das intervenções visando a correção da doença cardíaca estrutural ou funcional subjacente e o regime anticoagulante ideal requerem investigações adicionais.

As equipes multidisciplinares de Cardio-Oncologia estão numa posição privilegiada para continuar com esta missão, pois garantem uma abordagem verdadeiramente holística nesses pacientes desafiadores.

## References

[1] January CT, Wann LS, Calkins H, Chen LY, Cigarroa JE, Cleveland JC, Jr., et al. 2019 AHA/ACC/HRS Focused Update of the 2014 AHA/ACC/HRS Guideline for the Management of Patients With Atrial Fibrillation: A Report of the American College of Cardiology/American Heart Association Task Force on Clinical Practice Guidelines and the Heart Rhythm Society in Collaboration With the Society of Thoracic Surgeons. Circulation. 2019;140(2):e125-e151. doi: 10.1161/CIR.0000000000000665 10.1161/CIR.0000000000000665 30686041

[2] Hindricks G, Potpara T, Dagres N, Arbelo E, Bax JJ, Blomström-Lundqvist C, et al. 2020 ESC Guidelines for the diagnosis and management of atrial fibrillation developed in collaboration with the European Association of C.ardio-Thoracic Surgery (EACTS). Eur Heart J.2021;42(5):373-498. doi: 10.1093/eurheartj/ehaa612. 10.1093/eurheartj/ehaa612 32860505

[3] Heeringa J, van der Kuip DA, Hofman A, Kors JA, van Herpen G, Stricker BH, et al. Prevalence, incidence and lifetime risk of atrial fibrillation: the Rotterdam study. Eur Heart J. 2006;27(8):949-53. doi: 10.1093/eurheartj/ehi825 10.1093/eurheartj/ehi825 16527828

[4] Conen D, Wong JA, Sandhu RK, Cook NR, Lee IM, Buring JE, et al. Risk of Malignant Cancer Among Women With New-Onset Atrial Fibrillation. JAMA Cardiol. 2016;1(4):389-96. doi: 10.1001/jamacardio.2016.0280. 10.1001/jamacardio.2016.0280 PMC4957657 27438314

[5] Yuan M, Zhang Z, Tse G, Feng X, Korantzopoulos P, Letsas KP, et al. Association of Cancer and the Risk of Developing Atrial Fibrillation: A Systematic Review and Meta-Analysis. Cardiol Res Pract. 2019;2019:8985273. doi: 10.1155/2019/8985273. 10.1155/2019/8985273 PMC6487146 31110819

[6] Erichsen R, Christiansen CF, Mehnert F, Weiss NS, Baron JA, Sørensen HT. Colorectal cancer and risk of atrial fibrillation and flutter: a population-based case-control study. Intern Emerg Med. 2012;7(5):431-8. doi: 10.1007/s11739-011-0701-9. 10.1007/s11739-011-0701-9 21968511

[7] Vinter N, Christesen AMS, Fenger-Grøn M, Tjønneland A, Frost L. Atrial Fibrillation and Risk of Cancer: A Danish Population-Based Cohort Study. J Am Heart Assoc.2018;7(17):e009543. doi: 10.1161/JAHA.118.009543. 10.1161/JAHA.118.009543 PMC6201425 30371150

[8] Jakobsen CB, Lamberts M, Carlson N, Lock-Hansen M, Torp-Pedersen C, Gislason GH, et al. Incidence of atrial fibrillation in different major cancer subtypes: a Nationwide population-based 12 year follow up study. BMC Cancer. 2019;19(1):1105. doi: 10.1186/s12885-019-6314-9. 10.1186/s12885-019-6314-9 PMC6854796 31726997

[9] Ostenfeld EB, Erichsen R, Pedersen L, Farkas DK, Weiss NS, Sørensen HT. Atrial fibrillation as a marker of occult cancer. PloS One. 2014;9(8):e102861. doi: 10.1371/journal.pone.0102861. 10.1371/journal.pone.0102861 PMC4138009 25119880

[10] Pisters R, van Oostenbrugge RJ, Knottnerus IL, de Vos CB, Boreas A, Lodder J, et al. The likelihood of decreasing strokes in atrial fibrillation patients by strict application of guidelines. Europace.2010;12(6):779-84. doi: 10.1093/europace/euq080. 10.1093/europace/euq080 20348143

[11] Lateef N, Kapoor V, Ahsan MJ, Latif A, Ahmed U, Mirza M, et al. Atrial fibrillation and cancer; understanding the mysterious relationship through a systematic review. J Comm Community Hosp Intern Med Perspectiv. 2020;10(2):127-32. doi: 10.1080/20009666.2020.1726571. 10.1080/20009666.2020.1726571 PMC7425610 32850047

[12] Velagapudi P, Turagam MK, Kocheril AG. Atrial fibrillation in cancer patients: an underrecognized condition. Southern Med J. 2011;104(9):667-8. doi: 10.1097/SMJ.0b013e3182299e6c. 10.1097/SMJ.0b013e3182299e6c 21886091

[13] Mao L, Huang W, Zou P, Dang X, Zeng X. The unrecognized role of tumor suppressor genes in atrial fibrillation. Gene. 2018;642:26-31. doi: 10.1016/j.gene.2017.11.015. 10.1016/j.gene.2017.11.015 29126922

[14] Guzzetti S, Costantino G, Fundarò C. Systemic inflammation, atrial fibrillation, and cancer. Circulation. 2002;106(9):e40. doi: 10.1161/01.cir.0000028399.42411.13. 10.1161/01.cir.0000028399.42411.13 12196350

[15] Frustaci A, Chimenti C, Bellocci F, Morgante E, Russo MA, Maseri A. Histological substrate of atrial biopsies in patients with lone atrial fibrillation. Circulation. 1997;96(4):1180-4. doi: 10.1161/01.cir.96.4.1180. 10.1161/01.cir.96.4.1180 9286947

[16] Asselbergs FW, van den Berg MP, Diercks GF, van Gilst WH, van Veldhuisen DJ. C-reactive protein and microalbuminuria are associated with atrial fibrillation. Int J Cardiol. 2005;98(1):73-7. doi: 10.1016/j.ijcard.2003.12.028. 10.1016/j.ijcard.2003.12.028 15676170

[17] Marott SC, Nordestgaard BG, Zacho J, Friberg J, Jensen GB, Tybjaerg-Hansen A, et al. Does elevated C-reactive protein increase atrial fibrillation risk? A Mendelian randomization of 47,000 individuals from the general population. J Am Coll Cardiol. 2010;56(10):789-95. doi: 10.1016/j.jacc.2010.02.066. 10.1016/j.jacc.2010.02.066 20797493

[18] Hak Ł, Myśliwska J, Wieckiewicz J, Szyndler K, Siebert J, Rogowski J. Interleukin-2 as a predictor of early postoperative atrial fibrillation after cardiopulmonary bypass graft (CABG). J Interferon Cytokine Res. 2009;29(6):327-32. doi: 10.1089/jir.2008.0082.2906. 10.1089/jir.2008.0082.2906 19450160

[19] Marcus GM, Smith LM, Ordovas K, Scheinman MM, Kim AM, Badhwar N, et al. Intracardiac and extracardiac markers of inflammation during atrial fibrillation. Heart Rhythm. 2010;7(2):149-54. doi: 10.1016/j.hrthm.2009.10.004 10.1016/j.hrthm.2009.10.004 PMC2900773 20022819

[20] Li J, Solus J, Chen Q, Rho YH, Milne G, Stein CM, et al. Role of inflammation and oxidative stress in atrial fibrillation. Heart Rhythm. 2010;7(4):438-44. doi: 10.1016/j.hrthm.2009.12.009. 10.1016/j.hrthm.2009.12.009 PMC2843774 20153266

[21] Coussens LM, Werb Z. Inflammation and cancer. Nature. 2002;420(6917):860-7. doi: 10.1038/nature01322. 10.1038/nature01322 PMC2803035 12490959

[22] Multhoff G, Molls M, Radons J. Chronic inflammation in cancer development. Front Immunol. 2011;2:98. doi: 10.3389/fimmu.2011.00098 10.3389/fimmu.2011.00098 PMC3342348 22566887

[23] Farmakis D, Parissis J, Filippatos G. Insights into onco-cardiology: atrial fibrillation in cancer. J Am Coll Cardiol. 2014;63(10):945-53. doi: 10.1016/j.jacc.2013.11.026. 10.1016/j.jacc.2013.11.026 24361314

[24] Onaitis M, D’Amico T, Zhao Y, O’Brien S, Harpole D. Risk factors for atrial fibrillation after lung cancer surgery: analysis of the Society of Thoracic Surgeons general thoracic surgery database. Ann Thorac Surg.2010;90(2):368-74. doi: 10.1016/j.athoracsur.2010.03.100. 10.1016/j.athoracsur.2010.03.100 20667313

[25] Ojima T, Iwahashi M, Nakamori M, Nakamura M, Katsuda M, Iida T, et al. Atrial fibrillation after esophageal cancer surgery: an analysis of 207 consecutive patients. Surg Today. 2014;44(5):839-47. doi: 10.1007/s00595-013-0616-3 10.1007/s00595-013-0616-3 23674202

[26] Guzzetti S, Costantino G, Vernocchi A, Sada S, Fundarò C. First diagnosis of colorectal or breast cancer and prevalence of atrial fibrillation. emergency Intern Emerg Med. 2008;3(3):227-31. doi: 10.1007/s11739-008-0124-4. 10.1007/s11739-008-0124-4 18320149

[27] Guzzetti S, Costantino G, Sada S, Fundarò C. Colorectal cancer and atrial fibrillation: a case-control study. Am J Med. 2002;112(7):587-8. doi: 10.1016/s0002-9343(02)01029-x. 10.1016/s0002-9343(02)01029-x 12015256

[28] Leong DP, Caron F, Hillis C, Duan A, Healey JS, Fraser G, et al. The risk of atrial fibrillation with ibrutinib use: a systematic review and meta-analysis. Blood. 2016;128(1):138-40. doi: 10.1182/blood-2016-05-712828 10.1182/blood-2016-05-712828 27247135

[29] Shatzel JJ, Olson SR, Tao DL, McCarty OJT, Danilov AV, DeLoughery TG. Ibrutinib-associated bleeding: pathogenesis, management and risk reduction strategies. J Thromb Haemost. 2017;15(5):835-47. https://doi.org/10.1111/jth.13651 10.1111/jth.13651 PMC6152914 28182323

[30] Sharma R, Oni OA, Gupta K, Sharma M, Sharma R, Singh V, et al. Normalization of Testosterone Levels After Testosterone Replacement Therapy Is Associated With Decreased Incidence of Atrial Fibrillation. J Am Heart Assoc. 2017;6(5):835-47. doi: 10.1016/j.mayocpiqo.2017.05.003. 10.1161/JAHA.116.004880 PMC5524065 28487389

[31] Milliez P, Girerd X, Plouin PF, Blacher J, Safar ME, Mourad JJ. Evidence for an increased rate of cardiovascular events in patients with primary aldosteronism. J Am Coll Cardiol. 2005;45(8):1243-8. doi: 10.1016/j.jacc.2005.01.015. 10.1016/j.jacc.2005.01.015 15837256

[32] Martin Huertas R, Saavedra Serrano C, Perna C, Ferrer Gómez A, Alonso Gordoa T. Cardiac toxicity of immune-checkpoint inhibitors: a clinical case of nivolumab-induced myocarditis and review of the evidence and new challenges. Cancer Manag Med Res. 2019;11:4541-8. doi: 10.2147/CMAR.S185202. 10.2147/CMAR.S185202 PMC6529611 31191015

[33] Flaker GC, Belew K, Beckman K, Vidaillet H, Kron J, Safford R, et al. Asymptomatic atrial fibrillation: demographic features and prognostic information from the Atrial Fibrillation Follow-up Investigation of Rhythm Management (AFFIRM) study. Am Heart J. 2005;149(4):657-63. doi: 10.1016/j.ahj.2004.06.032. 10.1016/j.ahj.2004.06.032 15990749

[34] Boriani G, Laroche C, Diemberger I, Fantecchi E, Popescu MI, Rasmussen LH, et al. Asymptomatic atrial fibrillation: clinical correlates, management, and outcomes in the EORP-AF Pilot General Registry. Am J Med. 2015;128(5):509-18.e2. doi: 10.1016/j.amjmed.2014.11.026. 10.1016/j.amjmed.2014.11.026 25534423

[35] Lubitz SA, Yin X, McManus DD, Weng LC, Aparicio HJ, Walkey AJ, et al. Stroke as the Initial Manifestation of Atrial Fibrillation: The Framingham Heart Study. Stroke. 2017;48(2):490-2. DOI: 10.1016/j.amjmed.2014.11.026 10.1161/STROKEAHA.116.015071 PMC5262530 28082669

[36] Odutayo A, Wong CX, Hsiao AJ, Hopewell S, Altman DG, Emdin CA. Atrial fibrillation and risks of cardiovascular disease, renal disease, and death: systematic review and meta-analysis. BMJ. 2016;354:i4482. doi: 10.1136/bmj.i4482. 10.1136/bmj.i4482 27599725

[37] Friberg L, Hammar N, Pettersson H, Rosenqvist M. Increased mortality in paroxysmal atrial fibrillation: report from the Stockholm Cohort-Study of Atrial Fibrillation (SCAF). Eur Heart J. 2007;28(19):2346-53. doi: 10.1093/eurheartj/ehm308. 10.1093/eurheartj/ehm308 17670754

[38] Andersson T, Magnuson A, Bryngelsson IL, Frøbert O, Henriksson KM, Edvardsson N, et al. All-cause mortality in 272,186 patients hospitalized with incident atrial fibrillation 1995-2008: a Swedish nationwide long-term case-control study. Eur Heart J. 2013;34(14):1061-7. DOI: 10.1093/eurheartj/ehs469 10.1093/eurheartj/ehs469 PMC3618889 23321349

[39] Lee E, Choi EK, Han KD, Lee H, Choe WS, Lee SR, et al. Mortality and causes of death in patients with atrial fibrillation: A nationwide population-based study. PloS One. 2018;13(12):e0209687. DOI: 10.1371/journal.pone.0209687 10.1371/journal.pone.0209687 PMC6306259 30586468

[40] Ferreira C, Providência R, Ferreira MJ, Gonçalves LM. Atrial Fibrillation and Non-cardiovascular Diseases: A Systematic Review. Arq Bras Cardiol. 2015;105:519-26. doi: 10.5935/abc.20150142. 10.5935/abc.20150142 PMC4651411 26577719

[41] Imperatori A, Mariscalco G, Riganti G, Rotolo N, Conti V, Dominioni L. Atrial fibrillation after pulmonary lobectomy for lung cancer affects long-term survival in a prospective single-center study. J Cardiothor Surg. 2012;7:4. doi: 10.1186/1749-8090-7-4. 10.1186/1749-8090-7-4 PMC3287133 22233837

[42] Mairesse GH, Moran P, Van Gelder IC, Elsner C, Rosenqvist M, Mant J, et al. Screening for atrial fibrillation: a European Heart Rhythm Association (EHRA) consensus document endorsed by the Heart Rhythm Society (HRS), Asia Pacific Heart Rhythm Society (APHRS), and Sociedad Latinoamericana de Estimulación Cardíaca y Electrofisiología (SOLAECE). Europace. 2017;19(10):1589-623. doi: 10.1093/europace/eux177. 10.1093/europace/eux177 29048522

[43] Lowres N, Neubeck L, Salkeld G, Krass I, McLachlan AJ, Redfern J, et al. Feasibility and cost-effectiveness of stroke prevention through community screening for atrial fibrillation using iPhone ECG in pharmacies. The SEARCH-AF study. Thromb Haemost. 2014;111(6):1167-76. doi: 10.1160/TH14-03-0231 10.1160/TH14-03-0231 24687081

[44] Svennberg E, Engdahl J, Al-Khalili F, Friberg L, Frykman V, Rosenqvist M. Mass Screening for Untreated Atrial Fibrillation: The STROKESTOP Study. Circulation. 2015;131(25):2176-84. doi: 10.1161/CIRCULATIONAHA.114.014343. 10.1161/CIRCULATIONAHA.114.014343 25910800

[45] Chan PH, Wong CK, Poh YC, Pun L, Leung WW, Wong YF, et al. Diagnostic Performance of a Smartphone-Based Photoplethysmographic Application for Atrial Fibrillation Screening in a Primary Care Setting. J Am Heart J.2016;5(7):e003428. 10.1161/JAHA.116.003428 PMC5015379 27444506

[46] Perez MV, Mahaffey KW, Hedlin H, Rumsfeld JS, Garcia A, Ferris T, et al. Large-Scale Assessment of a Smartwatch to Identify Atrial Fibrillation. N Engl J Med. 2019;381(20):1909-17. doi: 10.1056/NEJMoa1901183. 10.1056/NEJMoa1901183 PMC8112605 31722151

[47] Fitzmaurice DA, Hobbs FD, Jowett S, Mant J, Murray ET, Holder R, et al. Screening versus routine practice in detection of atrial fibrillation in patients aged 65 or over: cluster randomised controlled trial. BMJ.2007;335(7616):383. doi: 10.1136/bmj.39280.660567.55. 10.1136/bmj.39280.660567.55 PMC1952508 17673732

[48] Halcox JPJ, Wareham K, Cardew A, Gilmore M, Barry JP, Phillips C, et al. Assessment of Remote Heart Rhythm Sampling Using the AliveCor Heart Monitor to Screen for Atrial Fibrillation: The REHEARSE-AF Study. Circulation. 2017;136(19):1784-94. doi: 10.1161/CIRCULATIONAHA.117.030583. 10.1161/CIRCULATIONAHA.117.030583 28851729

[49] Steinhubl SR, Waalen J, Edwards AM, Ariniello LM, Mehta RR, Ebner GS, et al. Effect of a Home-Based Wearable Continuous ECG Monitoring Patch on Detection of Undiagnosed Atrial Fibrillation: The mSToPS Randomized Clinical Trial. JAMA. 2018;320(2):146-55. doi: 10.1001/jama.2018.8102. 10.1001/jama.2018.8102 PMC6583518 29998336

[50] Sanna T, Diener HC, Passman RS, Di Lazzaro V, Bernstein RA, Morillo CA, et al. Cryptogenic stroke and underlying atrial fibrillation. N Engl Med.2014;370(26):2478-86. doi: 10.1056/NEJMoa1313600. 10.1056/NEJMoa1313600 24963567

[51] Gladstone DJ, Spring M, Dorian P, Panzov V, Thorpe KE, Hall J, et al. Atrial fibrillation in patients with cryptogenic stroke. N Engl J Med. 014;370(26):2467-77. doi: 10.1056/NEJMoa1311376. 10.1056/NEJMoa1311376 24963566

[52] Berkovitch A, Sabbag A, Segev S, Kivity S, Sidi Y, Goldenberg I, et al. CHADS-VASC SCORE and the risk of new onset atrial fibrillation among middle age adults. J Am Coll Cardiol. 2016;67(13 Suppl.):881.

[53] Wojszel ZB, Kasiukiewicz A, Swietek M, Swietek ML, Magnuszewski L. CHA2DS2-VASc score can guide the screening of atrial fibrillation - cross-sectional study in a geriatric ward. Clin Interv Aging.2019;14:879-87. doi: 10.2147/CIA.S206976. 10.2147/CIA.S206976 PMC6527793 31190774

[54] Engdahl J, Svennberg E, Friberg L, Al-Khalili F, Frykman V, Kemp Gudmundsdottir K, et al. Stepwise mass screening for atrial fibrillation using N-terminal pro b-type natriuretic peptide: the STROKESTOP II study design. Europace. 2016;19(2):297-302. doi: 10.2147/CIA.S206976. 10.1093/europace/euw319 28011798

[55] Ghazal F, Theobald H, Rosenqvist M, Al-Khalili F. Assessment of N-terminal pro-B-type natriuretic peptide level in screening for atrial fibrillation in primary health care. PloS One. 2019;14(2):e0212974. doi: 10.2147/CIA.S206976. 10.1371/journal.pone.0212974 PMC6391046 30807614

[56] Kemp Gudmundsdottir K, Fredriksson T, Svennberg E, Al-Khalili F, Friberg L, Frykman V, et al. Stepwise mass screening for atrial fibrillation using N-terminal B-type natriuretic peptide: the STROKESTOP II study. Europace. 2020;22(1):24-32. 10.1093/europace/euz255 PMC6945054 31790147

[57] Hart RG, Sharma M, Mundl H, Kasner SE, Bangdiwala SI, Berkowitz SD, et al. Rivaroxaban for Stroke Prevention after Embolic Stroke of Undetermined Source. NN Engl J Med. 2018;378(23):2191-201. doi: 10.1056/NEJMoa1802686. 10.1056/NEJMoa1802686 29766772

[58] Diener HC, Sacco RL, Easton JD, Granger CB, Bernstein RA, Uchiyama S, et al. Dabigatran for Prevention of Stroke after Embolic Stroke of Undetermined Source. N Engl J Med.2019;380(20):1906-17. doi: 10.1056/NEJMoa1813959 10.1056/NEJMoa1813959 31091372

[59] Chan PH, Wong CK, Pun L, Wong YF, Wong MM, Chu DW, et al. Diagnostic performance of an automatic blood pressure measurement device, Microlife WatchBP Home A, for atrial fibrillation screening in a real-world primary care setting. BMJ. 2017;7(6):e013685. doi: 10.1136/bmjopen-2016-013685. 10.1136/bmjopen-2016-013685 PMC5577883 28619766

[60] Freedman B, Camm J, Calkins H, Heally JS, Rosenqvist M, Wang J, et al. Screening for atrial fibrillation with ECG to reduce stroke. A report of the AF-SCREEN International Collaborators. Circulation.2017;135(19):1851-67. doi: 10.1161/CIRCULATIONAHA.116.026693. 10.1161/CIRCULATIONAHA.116.026693 28483832

[61] Diederichsen AC, Rasmussen LM, Søgaard R, Lambrechtsen J, Steffensen FH, Frost L, et al. The Danish Cardiovascular Screening Trial (DANCAVAS): study protocol for a randomized controlled trial. Trials. 2015;16:554. doi: 10.1186/s13063-015-1082-6. doi: 10.1186/s13063-015-1082-6. 10.1186/s13063-015-1082-6 PMC4670524 26637993

[62] Diederichsen SZ, Haugan KJ, Køber L, Højberg S, Brandes A, Kronborg C, et al. Atrial fibrillation detected by continuous electrocardiographic monitoring using implantable loop recorder to prevent stroke in individuals at risk (the LOOP study): Rationale and design of a large randomized controlled trial. Am Heart J. 2017;187 Am Heart J.2017;187:122-32. doi: 10.1016/j.ahj.2017.02.017. 10.1016/j.ahj.2017.02.017 28454796

[63] Benjamin EJ, Al-Khatib SM, Desvigne-Nickens P, Alonso A, Djoussé P, et al. J Am Heart Assoc.2021;10(16):e021566. doi: 10.1161/JAHA.121.021566 10.1161/JAHA.121.021566 PMC8475065 34351783

[64] Healey JS, Connolly SJ, Gold MR, Israel CW, Van Gelder IC, Capucci A, et al. Subclinical Atrial Fibrillation and the Risk of Stroke. N Engl J Med. 2012;366(2):120-9. doi: 10.1056/NEJMoa1105575. 10.1056/NEJMoa1105575 22236222

[65] Curry SJ, Krist AH, Owens DK, Barry MJ, Caughey AB, Davidson KW, et al. Screening for Atrial Fibrillation With Electrocardiography: US Preventive Services Task Force Recommendation Statement. JAMA.2018;320(5):478-84. doi: 10.1001/jama.2018.10321. 10.1001/jama.2018.10321 30088016

[66] Steinhubl SR, Mehta RR, Ebner GS, Ballesteros MM, Waalen J, Steinberg G, et al. Rationale and design of a home-based trial using wearable sensors to detect asymptomatic atrial fibrillation in a targeted population: The mHealth Screening To Prevent Strokes (mSToPS) trial. Am Heart J. 2016;175:77-85. doi: 10.1016/j.ahj.2016.02.011. 10.1016/j.ahj.2016.02.011 27179726

[67] Weber-Krüger M, Gelbrich G, Stahrenberg R, Liman J, Kermer P, Hamann GF, et al. Finding atrial fibrillation in stroke patients: Randomized evaluation of enhanced and prolonged Holter monitoring--Find-AF(RANDOMISED)-rationale and design. Am Heart J. 2014;168(4):438-45.e1. doi: 10.1016/j.ahj.2014.06.018. 10.1016/j.ahj.2014.06.018 25262252

[68] Roldán V, Marín F, Manzano-Fernández S, Gallego P, Vílchez JA, Valdés M, et al. The HAS-BLED score has better prediction accuracy for major bleeding than CHADS2 or CHA2DS2-VASc scores in anticoagulated patients with atrial fibrillation. J Am Coll Cardiol.2013;62(23):2199-204. doi: 10.1016/j.jacc.2013.08.1623. 10.1016/j.jacc.2013.08.1623 24055744

[69] Connolly SJ, Ezekowitz MD, Yusuf S, Eikelboom J, Oldgren J, Parekh A, et al. Dabigatran versus Warfarin in Patients with Atrial Fibrillation. N Engl J Med. 2009;361(12):1139-51. doi: 10.1056/NEJMoa0905561. 10.1056/NEJMoa0905561 19717844

[70] Patel MR, Mahaffey KW, Garg J, Pan G, Singer DE, Hacke W, et al. Rivaroxaban versus Warfarin in Nonvalvular Atrial Fibrillation. N Engl J Med. 2011;365(10):883-91. doi: 10.1056/NEJMoa1009638. 10.1056/NEJMoa1009638 21830957

[71] Granger CB, Alexander JH, McMurray JJV, Lopes RD, Hylek EM, Hanna M, et al. Apixaban versus Warfarin in Patients with Atrial Fibrillation. N Engl J Med. 2011;365(11):981-92. doi: 10.1056/NEJMoa1107039. 10.1056/NEJMoa1107039 21870978

[72] Giugliano RP, Ruff CT, Braunwald E, Murphy SA, Wiviott SD, Halperin JL, et al. Edoxaban versus Warfarin in Patients with Atrial Fibrillation. N Engl J Med. 2013;369(22):2093-104. doi: 10.1056/NEJMoa1310907. 10.1056/NEJMoa1310907 24251359

[73] Khorana AA, Francis CW, Culakova E, Kuderer NM, Lyman GH. Thromboembolism is a leading cause of death in cancer patients receiving outpatient chemotherapy. J Thromb Haemost. 2007;5(3):632-4. doi: 10.1111/j.1538-7836.2007.02374.x. 10.1111/j.1538-7836.2007.02374.x 17319909

[74] D’Souza M, Carlson N, Fosbøl E, Lamberts M, Smedegaard L, Nielsen D, et al. CHA(2)DS(2)-VASc score and risk of thromboembolism and bleeding in patients with atrial fibrillation and recent cancer. Eur J Prev Cardiol. 2018;25(6):651-8. doi: 10.1177/2047487318759858. 10.1177/2047487318759858 29482441

[75] Patell R, Gutierrez A, Rybicki L, Khorana AA. Usefulness of CHADS2 and CHA2DS2-VASc Scores for Stroke Prediction in Patients With Cancer and Atrial Fibrillation. Am J Cardiol. 2017;120(12):2182-6. doi: 10.1016/j.amjcard.2017.08.038 10.1016/j.amjcard.2017.08.038 29033049

[76] Hu YF, Liu CJ, Chang PM, Tsao HM, Lin YJ, Chang SL, et al. Incident thromboembolism and heart failure associated with new-onset atrial fibrillation in cancer patients. Int J Cardiol. 2013;165(2):355-7. doi: 10.1016/j.ijcard.2012.08.036. 10.1016/j.ijcard.2012.08.036 22989607

[77] Kamphuisen PW, Beyer-Westendorf J. Bleeding complications during anticoagulant treatment in patients with cancer. Thromb Res. 2014;133 (Suppl 2):S49-55. doi: 10.1016/S0049-3848(14)50009-6. 10.1016/S0049-3848(14)50009-6 24862146

[78] Melloni C, Shrader P, Carver J, Piccini JP, Thomas L, Fonarow GC, et al. Management and outcomes of patients with atrial fibrillation and a history of cancer: the ORBIT-AF registry. Eur Heart J Qual Care Clin Outcomes. 2017;3(3):192-7. doi: 10.1093/ehjqcco/qcx004. 10.1093/ehjqcco/qcx004 28838088

[79] Khorana AA, Soff GA, Kakkar AK, Vadhan-Raj S, Riess H, Wun T, et al. Rivaroxaban for Thromboprophylaxis in High-Risk Ambulatory Patients with Cancer. N Engl J Med. 2019;380(8):720-8. doi: 10.1056/NEJMoa1814630. 10.1056/NEJMoa1814630 30786186

[80] Carrier M, Abou-Nassar K, Mallick R, Tagalakis V, Shivakumar S, Schattner A, et al. Apixaban to Prevent Venous Thromboembolism in Patients with Cancer. N Engl J Med. 2018;380(8):711-9. doi: 10.1056/NEJMoa1814468. 10.1056/NEJMoa1814468 30511879

[81] Raskob GE, van Es N, Verhamme P, Carrier M, Di Nisio M, Garcia D, et al. Edoxaban for the Treatment of Cancer-Associated Venous Thromboembolism. N Engl J Med.2017; 378(7):615-24. doi: 10.1056/NEJMoa1711948. 10.1056/NEJMoa1711948 29231094

[82] Young AM, Marshall A, Thirlwall J, Chapman O, Lokare A, Hill C, et al. Comparison of an Oral Factor Xa Inhibitor With Low Molecular Weight Heparin in Patients With Cancer With Venous Thromboembolism: Results of a Randomized Trial (SELECT-D). J Clin Oncol. 2018;36(20):2017-23. doi: 10.1200/JCO.2018.78.8034. 10.1200/JCO.2018.78.8034 29746227

[83] Agnelli G, Becattini C, Meyer G, Muñoz A, Huisman MV, Connors JM, et al. Apixaban for the Treatment of Venous Thromboembolism Associated with Cancer. N Engl J Med. 2020;382(17):1599-607. doi: 10.1056/NEJMoa1915103. 10.1056/NEJMoa1915103 32223112

[84] Shah S, Norby FL, Datta YH, Lutsey PL, MacLehose RF, Chen LY, et al. Comparative effectiveness of direct oral anticoagulants and warfarin in patients with cancer and atrial fibrillation. Blood Adv.2018;2(3):200-9. doi: 10.1182/bloodadvances.2017010694. 10.1182/bloodadvances.2017010694 PMC5812321 29378726

[85] Melloni C, Dunning A, Granger CB, Thomas L, Khouri MG, Garcia DA, et al. Efficacy and Safety of Apixaban Versus Warfarin in Patients with Atrial Fibrillation and a History of Cancer: Insights from the ARISTOTLE Trial. Am J Med. 2017;130(12):1440-8.e1. doi: 10.1016/j.amjmed.2017.06.026. 10.1016/j.amjmed.2017.06.026 28739198

[86] Fanola CL, Ruff CT, Murphy SA, Jin J, Duggal A, Babilonia NA, et al. Efficacy and Safety of Edoxaban in Patients With Active Malignancy and Atrial Fibrillation: Analysis of the ENGAGE AF - TIMI 48 Trial. J Am Heart Assoc. 2018;7(16):e008987. doi: 10.1161/JAHA.118.008987. 10.1161/JAHA.118.008987 PMC6201390 30369307

[87] Deng Y, Tong Y, Deng Y, Zou L, Li S, Chen H. Non-Vitamin K Antagonist Oral Anticoagulants Versus Warfarin in Patients With Cancer and Atrial Fibrillation: A Systematic Review and Meta-Analysis. J Am Heart Assoc. 2019;8(14):e012540. doi: 10.1161/JAHA.119.012540. 10.1161/JAHA.119.012540 PMC6662149 31310583

[88] Holmes DR Jr., Doshi SK, Kar S, Price MJ, Sanchez JM, Sievert H, et al. Left Atrial Appendage Closure as an Alternative to Warfarin for Stroke Prevention in Atrial Fibrillation: A Patient-Level Meta-Analysis. J Am Coll Cardiol. 2015;65(24):2614-23. doi: 10.1016/j.jacc.2015.04.025. 10.1016/j.jacc.2015.04.025 26088300

[89] Reddy VY, Möbius-Winkler S, Miller MA, Neuzil P, Schuler G, Wiebe J, et al. Left atrial appendage closure with the Watchman device in patients with a contraindication for oral anticoagulation: the ASAP study (ASA Plavix Feasibility Study With Watchman Left Atrial Appendage Closure Technology). J Am Coll Cardiol. 2013;61(25):2551-6. doi: 10.1016/j.jacc.2013.03.035. 10.1016/j.jacc.2013.03.035 23583249

[90] Wyse DG, Waldo AL, DiMarco JP, Domanski MJ, Rosenberg Y, Schron EB, et al. A Comparison of Rate Control and Rhythm Control in Patients with Atrial Fibrillation. N Engl J Med. 2002;347(23):1825-33. doi: 10.1056/NEJMoa021328. 10.1056/NEJMoa021328 12466506

[91] Kirchhof P, Camm AJ, Goette A, Brandes A, Eckardt L, Elvan A, et al. Early Rhythm-Control Therapy in Patients with Atrial Fibrillation. N Engl J Med.2020; 383(14):1305-16. doi: 10.1056/NEJMoa2019422. 10.1056/NEJMoa2019422 32865375

[92] Van Gelder IC, Groenveld HF, Crijns HJGM, Tuininga YS, Tijssen JGP, Alings AM, et al. Lenient versus Strict Rate Control in Patients with Atrial Fibrillation. N Engl J Med. 2010;362(15):1363-73. doi: 10.1056/NEJMoa1001337. 10.1056/NEJMoa1001337 20231232

[93] Steffel J, Verhamme P, Potpara TS, Albaladejo P, Antz M, Desteghe L, et al. The 2018 European Heart Rhythm Association Practical Guide on the use of non-vitamin K antagonist oral anticoagulants in patients with atrial fibrillation. Eur Heart J. 2018;39(16):1330-93. doi: 10.1093/eurheartj/ehy136. 10.1093/eurheartj/ehy136 29562325

[94] López-Fernández T, Martín-García A, Roldán Rabadán I, Mitroi C, Mazón Ramos P, Díez-Villanueva P, et al. Atrial Fibrillation in Active Cancer Patients: Expert Position Paper and Recommendatio. Rev Esp Cardiol. 2019;72(9):749-59. doi: 10.1016/j.rec.2019.03.019. 10.1016/j.rec.2019.03.019 31405794

[95] Siontis KC, Zhang X, Eckard A, Bhave N, Schaubel DE, He K, et al. Outcomes Associated With Apixaban Use in Patients With End-Stage Kidney Disease and Atrial Fibrillation in the United States. Circulation. 2018;138(15):1519-29. doi: 10.1161/CIRCULATIONAHA.118.035418. doi: 10.1002/rth2.12111. 10.1161/CIRCULATIONAHA.118.035418 PMC6202193 29954737

[96] Samuelson Bannow BR, Lee AYY, Khorana AA, Zwicker JI, Noble S, Ay C, et al. Management of anticoagulation for cancer-associated thrombosis in patients with thrombocytopenia: A systematic review. Res Pract Thromb Haemost.2018;2(4):644-69.. 2018;2(4):664-9. doi: 10.1002/rth2.12111. 10.1002/rth2.12111 PMC6178713 30349884

[97] Chung MK, Eckhardt LL, Chen LY, Ahmed HM, Gopinathannair R, Joglar JA, et al. Lifestyle and Risk Factor Modification for Reduction of Atrial Fibrillation: A Scientific Statement From the American Heart Association. Circulation. 2020;141(16):e750-e72. doi: 10.1161/CIR.0000000000000748. 10.1161/CIR.0000000000000748 32148086

[98] Gilchrist SC, Barac A, Ades PA, Alfano CM, Franklin BA, Jones LW, et al. Cardio-Oncology Rehabilitation to Manage Cardiovascular Outcomes in Cancer Patients and Survivors: A Scientific Statement From the American Heart Association. Circulation. 2019;139(21):e997-e1012. doi: 10.1161/CIR.0000000000000679. 10.1161/CIR.0000000000000679 PMC7603804 30955352

[99] Cormie P, Zopf EM, Zhang X, Schmitz KH. The Impact of Exercise on Cancer Mortality, Recurrence, and Treatment-Related Adverse Effects. Epidemiol Rev.2017;39(1):71-92. doi: 10.1093/epirev/mxx007. 10.1093/epirev/mxx007 28453622

[100] Attia ZI, Noseworthy PA, Lopez-Jimenez F, Asirvatham SJ, Deshmukh AJ, Gersh BJ, et al. An artificial intelligence-enabled ECG algorithm for the identification of patients with atrial fibrillation during sinus rhythm: a retrospective analysis of outcome prediction. Lancet. (London, England). 2019;394(10201):861-7. doi: 10.1016/S0140-6736(19)31721-0 10.1016/S0140-6736(19)31721-0 31378392

[101] Kawakami H, Ramkumar S, Nolan M, Wright L, Yang H, Negishi K, et al. Left Atrial Mechanical Dispersion Assessed by Strain Echocardiography as an Independent Predictor of New-Onset Atrial Fibrillation: A Case-Control Study. J Am Soc Echocardiogr. 2019;32(10):1268-76.e3. DOI: 10.1016/j.echo.2019.06.002 10.1016/j.echo.2019.06.002 31466848

[102] Andreasen L, Bertelsen L, Ghouse J, Lundegaard PR, Ahlberg G, Refsgaard L, et al. Early-onset atrial fibrillation patients show reduced left ventricular ejection fraction and increased atrial fibrosis. Sci Rep. 2020;10(1):10039. doi: 10.1038/s41598-020-66671-w. 10.1038/s41598-020-66671-w PMC7308347 32572052

[103] Habibi M, Lima JA, Khurram IM, Zimmerman SL, Zipunnikov V, Fukumoto K, et al. Association of left atrial function and left atrial enhancement in patients with atrial fibrillation: cardiac magnetic resonance study. Circ Cardiovasc Imaging. 2015;8(2):e002769. doi: 10.1038/s41598-020-66671-w. 10.1161/CIRCIMAGING.114.002769 PMC4319560 25652181

[104] Christophersen IE, Rienstra M, Roselli C, Yin X, Geelhoed B, Barnard J, et al. Large-scale analyses of common and rare variants identify 12 new loci associated with atrial fibrillation. Nat Genet. 2017;49(6):946-52. doi: 10.1038/ng.3843. 10.1038/ng.3843 PMC5585859 28416818

[105] Lyon AR, Dent S, Stanway S, Earl H, Brezden-Masley C, Cohen-Solal A, et al. Baseline cardiovascular risk assessment in cancer patients scheduled to receive cardiotoxic cancer therapies: a position statement and new risk assessment tools from the Cardio-Oncology Study Group of the Heart Failure Association of the European Society of Cardiology in collaboration with the International Cardio-Oncology Society. Eur J Heart Fail. 2020;22(11):1945-60. doi: 10.1002/ejhf.1920 10.1002/ejhf.1920 PMC8019326 32463967

