# Supplementary material for: Rastreamento, Diagnóstico e Manejo da Fibrilação Atrial em Pacientes com Câncer: Evidências Atuais e Perspectivas Futuras
Source: Arq Bras Cardiol. 2022 Jul 29;119(2):328–41. [Article in Portuguese] doi: 10.36660/abc.20201362 (PMC9363048; doi:10.36660/abc.20201362)
Supplement: Translation

# Translation: Screening, Diagnosis and Management of Atrial Fibrillation in Cancer Patients: Current Evidence and Future Perspectives

**Authors:** Pedro Gonçalves-Teixeira, Telma Costa, Isabel Fragoso, Diogo Ferreira, Mariana Brandão, Adelino Leite-Moreira, Francisco Sampaio, José Ribeiro, Ricardo Fontes-Carvalho

**Affiliations:** 1 Gaia Portugal Departamento de Cardiologia, Vila Nova de Gaia Hospital Center, Gaia – Portugal; 2 Porto Portugal Departamento de Fisiologia, Faculdade de Medicina, Universidade do Porto, Porto – Portugal; 3 Gaia Portugal Clínica Cardio-Oncológica, Centro Hospitalar Vila Nova de Gaia, Gaia – Portugal; 4 Gaia Portugal Departamento de Oncologia, Centro Hospitalar Vila Nova de Gaia, Gaia – Portugal; 5 Arazede Portugal Unidade de Atenção Primária à Saúde Aracetti, Arazede – Portugal; 6 Porto Portugal Departamento de Cirurgia Cardiotorácica, Hospital Universitário São João, Porto – Portugal; 7 Porto Portugal Unidade de Pesquisa Cardiovascular (UnIC), Faculdade de Medicina, Universidade do Porto, Porto – Portugal

**Keywords:** Atrial Fibrillation, Neoplasms, Cardiotoxicity, Mass Screening, Cardio-Oncology, Arrhythmias, Cardiac, Anticoagulants, Blood Coagulation

## Abstract

Atrial fibrillation (AF) is the most common sustained cardiac arrhythmia in the general population, carrying a high morbimortality burden, and this also holds true in cancer patients. The association between AF and cancer goes even further, with some studies suggesting that AF can be a marker of occult cancer. There is, however, a remarkable paucity of data concerning specific challenges of AF management in cancer patients. AF prompt recognition and management in this special population can lessen the arrhythmia-related morbidity and have an important prognostic benefit. This review will focus on current AF diagnosis and management challenges in cancer patients, with special emphasis on AF screening strategies and devices, and anticoagulation therapy with non-vitamin K antagonist oral anti-coagulants (NOACs) for thromboembolic prevention in these patients. Some insights concerning future perspectives for AF prevention, diagnosis, and treatment in this special population will also be addressed.

## Introduction

Cardio-oncology has emerged as a key clinical field in the management of cancer patients, over the past decade. Cardio-oncology clinics now provide truly patient-centered clinical care and has proved useful in the prevention of cancer therapy-related cardiovascular toxicity.

Traditionally, oncology clinics were limited to the awareness of potential cardiomyocyte toxicity and risk of subsequent heart failure. We now have an ever more matured view of the varied cancer therapy-related cardiotoxicity. This includes a broad spectrum of inflammatory, thromboembolic and arrhythmic complications.

### AF burden

Atrial Fibrillation (AF) is recognized as the most common sustained cardiac arrhythmia, with a prevalence of approximately 0.5 to 2% of the general population. Patients with AF have a five-fold increased risk of stroke and a three-fold increased risk of heart failure. Furthermore, AF is an independent predictor of cardiovascular morbidity and mortality.^1,2^

Factors predisposing to AF development include aging (with the prevalence of AF being as high as 10% in patients over 80 years old),^3^ cardiovascular disorders such as hypertension, valvular heart disease, heart failure, pulmonary hypertension, and a variety of non-cardiovascular comorbidities such as diabetes, chronic pulmonary disease, obstructive sleep apnea, chronic kidney disease, thyroid dysfunction, inflammatory bowel disease, amongst others.

The association between AF and cancer has long been recognized and is somewhat expected based on the increasing prevalence of cancer with aging, and the high frequency of comorbidities predisposing to AF in cancer patients.

Several population-based cohort studies showed the remarkable, bidirectional association between these entities. A recent meta-analysis showed that the rate of cancer diagnosis was three times higher in the first 3 months following AF diagnosis. Conversely, the risk of AF was particularly increased in the first 3 months after cancer diagnosis (OR 7.62, CI 3.08 to 18.88).^4,5^ Additionally, in a large population-based case-control study with 28,833 AF cases, 0.59% were diagnosed with colorectal cancer in the 90 days before AF diagnosis, compared with only 0.05% of the controls.^6^ Another cohort study also found that AF was associated with a higher incidence rate of cancer diagnosis in the next two decades of follow-up, and, again, this holds particularly true within 90 days after the diagnosis of AF. In this 90-day period men had an approximately 3-fold higher risk of having a cancer diagnosis, while women had a 4-fold higher risk.^7^ In a recently published observational study including 4,324,545 individuals, of which 316,040 had a cancer diagnosis, AF remained independently associated with all major cancer subtypes.^8^ The overall AF prevalence was 1.74% among cancer patients *vs*. 0.37% in the general population, and this difference increased with age. The strength of the association declined over time from the cancer diagnosis but remained significant even after 5 years (incidence rate ratio of 3.4 from day 0 to 90, and 1.1 from 2 to 5 years from cancer diagnosis). Another nationwide cohort study concluded that AF was strongly associated with metastatic cancer.^9^

It is known that AF can be an asymptomatic condition, especially in the elderly. The frequent paroxysmal nature of AF further complicates its early recognition. Studies have demonstrated that up to 45% of all AF-related strokes occurred in patients with asymptomatic and unknown AF.^10^ The significant risk for thromboembolic complications posed by AF is thought to be even greater in cancer patients, in whom a procoagulant state usually prevails.

Screening and searching for AF may have a potential role in preventing complications if adequate treatment is prescribed early.

On the other hand, as the association between AF and cancer goes even further, some studies suggest that AF can be a marker of occult cancer. The authors of a meta-analysis comprising 5 population-based observational studies including more than 5,500,000 patients recommended that patients with new-onset AF should be screened for occult cancer.^5^ This is, at present, highly controversial and has been contradicted by others.^7,11^

This review will focus on current AF diagnosis and management challenges in cancer patients, with special emphasis on AF screening, and anticoagulation therapy for thromboembolic stroke prevention in these patients. Some insights concerning future perspectives for AF prevention, identification, and treatment in this special population, will also be provided.

### AF and Cancer: proposed pathophysiological links

Multiple pathophysiological links have been proposed to explain the strong association between the two entities (Figure 1).

**Figure 1:** The multifactorial, bidirectional, interplay between cancer and atrial fibrillation. See text for further details.

The existence of shared risk factors for cancer and AF – such as preexisting cardiovascular disease, aging, obesity, diabetes, alcohol consumption and smoking – may explain a significant proportion of this epidemiological link.

Moreover, cancer patients frequently experience pain, hypoxia, electrolyte abnormalities and malnutrition, all of which can prompt several autonomic and endocrine-metabolic abnormalities contributing to AF.^12^

At the atria level, primary or metastatic tumor growth can elicit local compression or invasion, both potentially triggering AF.

It has been suggested that cancer increases the incidence of AF through the abnormal production of thyroid hormones-like peptides.^13^ A variety of paraneoplastic syndromes may ultimately lead to endocrine or metabolic derangements and set the stage for AF development. Other auto-immune mechanisms involving targeting of atrial tissue have been postulated.^14^

Occult undiagnosed cancer, with its accompanying altered autonomic tone and a pro-inflammatory state, may precede AF and explain, at least in part, the association. In some of these cases, anticoagulation therapy may unmask the neoplastic disorder by promoting tumor-related bleeding events. Also, being more closely exposed to medical examination and diagnostic tests, recently diagnosed cancer patients have higher probability of new-onset AF diagnosis.

There is a large amount of evidence linking AF to inflammatory disorders. The high prevalence of AF in the postoperative period and in the acute stages of acute myocardial infarction (AMI) or myocarditis, provide a valuable insight into the relationship between AF and inflammation. Histological studies further explored this, with AF patients showing inflammatory cell infiltrates in their right atrial endocardium, which was not observed in controls.^15^ Several studies evaluated inflammatory biomarkers in this context, showing C-reactive protein (CRP),^16,17^ interleukin 2 (IL-2),^18^ interleukin 6 (IL-6),^19^ tumor necrosis factor α (TNF-α) and monocyte chemoattractant protein 1 (MCP-1)^20^ to be significantly elevated in AF patients when compared to controls. The association between cancer and inflammation, being notably robust,^21,22^ allows the hypothesis that inflammation is probably a common substrate for AF and cancer in some patients.^23^

AF is frequently seen following surgical therapy for cancer, and this is particularly evident after pulmonary resection for lung cancer, with a large observational study showing a prevalence of 12.6%.^24^ This was also documented following surgery for esophageal, colorectal and breast cancers.^25-27^

Finally, several widely used anticancer drugs have been associated with an increased risk of incident AF (Table 1). A renewed interest in this field arose following the first reports of ibrutinib-related AF, a tyrosine-kinase inhibitor (TKi) used in patients with chronic lymphocytic leukemia, mantle cell lymphoma, and other hematological malignancies. The incidence of AF in patients undergoing ibrutinib therapy ranged from 3% to 16%.^28^ The unique antiplatelet effects of ibrutinib, which appears to inhibit the initial steps of platelet adhesion and activation,^29^ may pose therapeutic challenges when a decision must be reached about anticoagulation. It has been suggested that androgen deprivation therapy used to treat prostate cancer may lead to higher incidence of AF, possibly related to hormonotherapy-related hypogonadism.^30^ This risk was more pronounced with abiraterone, a drug that also blocks CYP17 enzymes, and thus can cause hypermineralocortisolism, promoting hypokalemia and AF.^31^ More recently, immune checkpoint inhibitors (ICI), have also been linked to new-onset AF because of their propension to cause myocardial and pericardial inflammation through auto-immune mechanisms.^32^ Other autoimmune side effects of ICIs, such as thyroiditis, may predispose to AF development as well.

**Table 1:** Reported frequency of cancer therapy-induced atrial fibrillation

Therapeutic class	Drug agent	Reported frequency of AF
Alkylating agents	Anthracyclines	0.55 – 10.3%
	Melphalan	10.8 – 33%
	Busulfan	6.4%
	Cyclophosphamide	2%
Antimetabolites	5-Fluorouracil	5%
	Capecitabine	0.5 – 1.1%
	Gemcitabine	0-8.1% (*)
Taxanes	Paclitaxel	0.18 - 1%
IImmunomodulators	Talidomide	4.7%
	Lenalidomide	4.6 - 7%
Platinum derivates	Cisplatin	10-32%
Tyrosine kinase inhibitors	Ibrutinib (BTK)	3-16%
	Nilotinib (BCR-ABL1)	0.8%
	Ponatinib (BCR-ABL1)	3-7%
	Vemurafenib (BRAF)	1.5%
	Imatinib (BCR-ABL1)	0.55 – 33%
	Dasatinib (BCR-ABL1)	5.6%
	Sorafenib (VEGFR)	5.1% (**)
Proteasome inhibitors	Bortezomib	2.2%
	Carfilzomib	3.2 – 3.8%
Monoclonal antibodies	Trastuzumab (HER2/ERBB2)	1.2%
	Bevacizumab (VEFG)	2.2%
	Cetuximab (EGFR/HER1)	4.8%
	Alentuzumab (CD52)	1.2%
	Rituximab (CD20)	1%
Other	Interleukin 2	4.3 – 8%
ICIs	Nivolumab (anti-PD1)	13%
	Pembrolizumab (anti-PD1)	
	Ipilimumab (anti-CTLA4)	
CAR-T cell therapy		2.2%
Hormonotherapy	Degarelix	2%
	Abiraterone	1 – 5%
Radiation therapy		0.5 –3.2%

(*) AF incidence of 0% when used alone, 8% when associated with vinorelbine.

(**) The reported prevalence was found in association with 5-FU, in a phase II study. It is noteworthy to recall that this association is not currently used in daily clinical practice.

Chest radiotherapy is associated with myocardial fibrosis, potentially causing a restrictive cardiomyopathy over the long term, and the associated filling pressure elevation favors AF development. Enhanced myocardial fibrosis at the atria level may set the stage for subsequent mechanical and/or electrical remodeling, ultimately causing AF.

It must be acknowledged, however, that the real incidence of cancer therapy-related AF is likely to be underestimated, as routine rhythm monitoring is seldom performed or comprises only a single recording of a 12-lead ECG.

### The rationale for AF screening

AF is not infrequently an asymptomatic condition, and the risk of stroke or death was found to be similar between symptomatic AF and silent AF.^33,34^ Up to 5% of individuals with AF have a stroke as the initial clinical manifestation of their arrhythmia.^35^ This may represent near one-third of all AF-related strokes. AF is associated with increased mortality risk in the general population,^36-39^ and this has also proved to be true in cancer patients.^40,41^

Prevention of thromboembolic stroke due to an early introduction of oral anticoagulation in patients at risk is perhaps the most plausible benefit of AF screening programs.^42^ Other proposed theoretical benefits of early AF recognition and management include reduction of AF-related morbidity and hospitalizations, and reduction of AF-related mortality.

The added value of opportunistic / systematic screening *versus* standard of care to detect silent AF in the general population is well established, and the rates of newly diagnosed AF ranged from 0.5 to 3.9% in most studies.^43-49^ The increasing yield of screening programs seems to be more intimately related to the screened population and the duration of screening, rather than specific devices / test characteristics.

Factors such as age,^44^ previous history of thromboembolic stroke,^50,51^ CHA2DS2-VASc score,^52,53^ and NT-proBNP levels,^54,55^ have been proposed as potentially useful to optimize the “number needed to screen” of such programs, possibly allowing for improved net clinical benefit and cost-effectiveness.

Interestingly, CHA2DS2-VASc score not only predicts stroke risk among patients with known AF, but also performs fairly well when predicting newly diagnosed AF. This may be useful as a gatekeeper for screening programs, not only (1) helping to select those patients with higher pre-test probability for silent AF, but also because (2) it warrants all detected cases will derive clinical benefit from oral anticoagulant (OAC) prescription.

The clinical trial STROKESTOP included 75- and 76-year-old individuals, thereby selecting participants with a CHA2DS2-VASc score of at least 2 points (age >75). Previously unknown AF was found in 0.5% of the screened population in their first ECG, whereas intermittent ECG recordings increased new AF detection by 4-fold.^44^

The STROKESTOP II study also added the use of NT-proBNP, in a stepwise strategy for AF screening in 75- and 76-year-old individuals. The high-risk group (NT-proBNP ≥125 ng/L) was offered extended ECG-screening, whereas the low-risk group performed only one single-lead ECG recording. In the high-risk group 4.4% had newly diagnosed AF.^56^

Even in cohorts at higher risk for thromboembolic stroke (i.e. those with previous embolic stroke of undetermined source), empirical treatment with OAC failed to demonstrate a reduction in recurrent stroke. This reinforces the importance of effective AF documentation prior to the implementation of such therapies,^57,58^ even in high prevalence and high-risk cohorts, such as cancer patients. In patients with documented AF, OAC therapy reduced stroke rates by two-thirds.^50^

### Strategies for AF screening

Several methods are available for AF screening (Figure 2). The simplest method for AF screening is pulse taking, which provides good sensitivity but only modest specificity (reported range of 65–91%). Other approaches include automated blood pressure devices (those able to perform oscillometric analysis),^59^ non-invasive devices for a single-lead ECG registration, and cardiac rhythm monitoring patches.

**Figure 2:** Several methods are available for outpatient atrial fibrillation screening. BP: blood pressure. CIED: cardiac implantable electronic device. ICM: implantable cardiac monitor

More recently, smartphone and smartwatch-based ambulatory monitoring introduced the ability for patient-activated monitoring without the need for wearable devices, and for indefinite periods. Such a smartwatch device showed promising results in a study with 419,000 participants, concerning mass-screening for AF. Irregular rhythm patterns were detected in 0.52% of participants, and this prompted subsequent confirmation with an electrocardiography (ECG) patch. The positive predictive value of the irregular rhythms detected by the smartwatch as possible AF was 0.71. It must be noted, however, the unfavorable age profile of the enrolled individuals, which were mostly young (52% were younger than 40 years and only 6% were 65 or older).^46^

Artificial intelligence-based rhythm analysis is frequently dependent on heterogeneous algorithms and, therefore, subsequent validation of findings is needed. This applies not only to plethysmography analysis for pulse wave irregularities but also for single-lead ECG generation of some devices, whose diagnostic accuracy does not yet replace human judgement. This may represent a challenge for healthcare systems, potentially leading to human resources’ shortness, since the great amount of data generated by these devices ultimately requires validation.

To date, randomized trials of AF screening have not demonstrated a reduction in stroke or other hard outcomes. It must be acknowledged, however, that none of these trials was adequately powered to demonstrate such an effect. Several trials are currently ongoing, aiming to give insights into this important topic (SAFER,^60^ DANCANVAS,^61^ LOOP,^62^ GUARD-AF^63^).

Two important drawbacks have been pointed out regarding AF screening strategies. The first one concerns the risk of false positive results and potential for increased bleeding risk in patients in which OAC does not bring clinical benefit. The expected psychological consequences of a false positive result, concerning anxiety levels and diminished quality of life, may have redoubled their importance in oncologic patients. The second emphasizes the uncertain clinical significance of short episodes of AF documented with prolonged screening modalities. In fact, these short-lasting arrythmia episodes may not represent an increased risk of thromboembolic events.^64^

Following new-onset AF detection with whichever screening strategy used, it must be stressed, nevertheless, that ECG confirmation of AF is still mandatory in the guidelines.^2^

### Current recommendations for AF screening

The European Society of Cardiology (ESC) recommends opportunistic screening for AF by pulse taking or ECG rhythm strip in patients aged >65 years-old, with a Class of Recommendation (COR) I and a Level of Evidence (LOE) B.^2^ According to the same recommendations, systematic ECG screening may be considered to detect AF in patients aged 75 years or older, or those at high stroke risk (COR IIb, LOE B). A position paper from the European Heart Rhythm Association (EHRA) adds that screening for AF is advised in high-risk populations, because of its cost-effectiveness.^42^

In contrast, the United States Preventive Services Task Force states that the current evidence is insufficient to assess the balance of benefits and harms of screening for AF with electrocardiography.^65^

Despite the high burden of AF in cancer patients, there are no specific recommendations regarding AF screening in these patients.

### AF screening in cancer patients: what is the evidence?

There is an astonishing paucity of data concerning AF screening in cancer patients. Moreover, current malignancy and/or chemotherapy or radiotherapy exposure were considered the exclusion criteria in some trials on AF screening.^62,66,67^

Intriguingly, most of AF screening studies do not even report cancer prevalence when it comes to the screened population characterization. Among the few studies that report cancer prevalence at baseline, no clear description exists regarding the rate of newly identified AF and/or “number needed to screen” in those patients.

A national cross-sectional study from Ireland randomly screened 2,200 patients aged 70 years and over using a three-lead ECG monitor in a primary care setting. The incident rate of newly diagnosed AF was 1.2%. This study reported a lung cancer prevalence of 0.3% in the overall screened population, but once again, no data on incident rate for newly identified AF is available for those patients.

### Management of AF in cancer patients

The overall principles of AF prevention and treatment, and general management recommendations, also apply to cancer patients. For the sake of consistency, the author’s will follow the guideline-recommended “ABC” approach to AF treatment (A: avoid stroke, anticoagulation; B: better symptom management, including patient-shared decisions on rate or rhythm control strategies; C: cardiovascular and comorbidity risk reduction). We also address some cancer patients’ particularities that deserve consideration.

#### Antithrombotic regimen

In AF patients from the general population, the ischemic stroke risk is stratified with satisfactory precision by CHA2DS2-VASc score, and patients with a score ≥1 (except for female gender alone) are considered to have favorable risk/benefit under OAC therapy.^2^ This must be balanced alongside the bleeding risk in each patient. The HAS-BLED score has been proposed for bleeding risk assessment in the general population.^68^ The HEMORR2HAGES risk assessment scale has the unique feature of including cancer as a risk factor for bleeding in AF, although it lacks external validation. Risk factor modification is of utmost importance to minimize bleeding risk. Apart from their suboptimal performance and discriminatory capacity, the numerous bleeding-risk scores available have the merit of highlighting such modifiable risk factors.

The OAC therapy reduces the risk of ischemic stroke rate by roughly 60%. Several landmark clinical trials have highlighted the superior safety profile of NOACs vs. VKAs with a comparable efficacy in the general population.^69-72^ However, these studies directly (precluding patients undergoing active chemotherapy/radiation therapy) or indirectly (not allowing the enrollment of individuals with an expected survival <12 months) excluded active cancer patients.

Thrombotic events are the second leading cause of mortality in cancer patients.^73^ However, cancer and many of its thrombotic-risk features are not incorporated into the CHA2DS2-VASc score calculation. Additionally, cancer-associated bleeding risk may theoretically shift the “net clinical benefit point” of OAC in these patients towards a higher CHA2DS2-VASc score (Figure 3).

**Figure 3:** Cancer patients with AF are at simultaneously high thrombotic and bleeding risk. Patient factors, as well as tumor-specific risks and cancer therapeutics adverse effects pose additional challenges. The indication for anticoagulation in these patients must be individualized, and several factors, not included in classic risk scores, should be considered. (*) Vinca alkaloids, alkylating agents, monoclonal antibodies (aflibercept, bevacizumab, ramucirumab, trastuzumab emtansine), antiestrogens, antimetabolites (pentostatin), anthracyclines, bleomycin, campothecins, carfilzomib, epipodophyllotoxins, ibrutinib, BCR-ABL, BRAF, and VEGF/VEGFR inhibitors, interleukins, L-asparaginase, ruxolitinib, taxanes, temozolomide, cyclophosphamide, ifosfamide, megestrol, tamoxifen. (**) CHA2DS2-VASc score is a strong predictor of thromboembolic events in patients with previously known AF but performed poorly for stroke risk prediction in those with cancer and newly diagnosed AF. See text for further details. (***) Alkylating agents (carboplatin, cyclophosphamide, cisplatin, estramustine, oxaliplatin, temozolomide), gonadotropin-releasing hormone analogs, antiandrogens, monoclonal antibodies (aflibercept, bevacizumab, cetuximab, panitumumab), anthracyclines, antimetabolites (capecitabine, 5-fluorouracil, gemcitabine, methotrexate, pentostatin), immunomodulators (lenalidomide, pomalidomide, thalidomide), aromatase inhibitors, bleomycin, protein kinase inhibitors (axitinib, lenvatinib, pazopanib, sorafenib, sunitinib), mTOR inhibitors, proteosome inhibitors (carfilzomib), irinotecan, taxanes, tasonermin, tretinoin, megestrol, progestogens, raloxifene, tamoxifen, vinflunine, vorinostat, erythropoiesis-stimulating agents and granulocyte colony-stimulating factors.

Conflicting analysis have been made concerning the CHADS2 and CHA2DS2-VASc scores performance in cancer patients with AF. In a study including over 120,000 patients, those with cancer and a low CHA2DS2-VASc score (0–1) had a higher risk of stroke than noncancer patients, but in those with a score ≥2, the stroke risk was similar between cancer and noncancer patients.^74^

In a study comprising roughly 2000 patients, the CHADS2 score was more predictive of increased stroke risk in patients with cancer and pre-existing AF (each point increase was associated with a nearly 40% greater risk of stroke) than the CHA2DS2-VASc.^75^ In the same study, notwithstanding, both scores accurately predicted the risk of stroke and survival. Intriguingly, the CHADS2 score lacked power to predict thromboembolism in cancer patients with new-onset AF in another study.^76^

On the other hand, patients with recently diagnosed cancer were at greater risk of bleeding, irrespective of the CHA2DS2-VASc score.^74^ Cancer patients have a noticeable higher risk for bleeding events, either due to malignancy location, cancer surgery, thrombocytopenia, platelet dysfunction, chemotherapeutic agents, radiation therapy, iatrogenic and/or tumor-related kidney or liver failure, bone marrow suppression (by the neoplastic disorder or cancer-related therapeutics), disseminated intravascular coagulation or hyperfibrinolysis in specific subsets, mucositis, and acquired von Willebrand syndrome. In the Riete registry,^77^ prior bleeding, creatinine clearance <30 mL/min, immobility ≥4 days and metastatic disease were the most important predictors of major bleeding in cancer patients undergoing anticoagulation therapy.

In a large registry data analysis, cancer patients had a two- to six-fold increase in the bleeding risk compared with patients without cancer.^78^ Ischemic stroke rate was, however, comparable.

Evidence from randomized clinical trials comparing NOACs to either vitamin K antagonist (VKA) or low-molecular weight heparin (LMWH) for thromboembolic prevention in cancer patients with AF is not available at the present date.

Several RCTs recently emphasized NOAC’s efficacy and safety profile for venous thromboembolism prophylaxis^79,80^ and treatment^81-83^ in cancer patients, compared to low molecular weight heparins (LMWH). In all these studies, the minor bleeding risk was greater with NOAC *versus* LMWH (driven by a higher rate of gastrointestinal bleeding). The major bleeding risk was similar between the two drug classes in some studies (Caravaggio^83^ and SELECT-D^82^), but an increased risk with NOAC use was observed in one trial (Hokusai VTE Cancer^81^). To some extent, cautious extrapolation can be made from these trials, but the unique thromboembolic pathophysiology in AF patients deserves dedicated trials.

Recent observational data from a cohort of 16,096 patients with AF and cancer suggest NOACs may be at least as effective as warfarin for the prevention of ischemic stroke and have a safer bleeding profile.^84^

A summary of various subanalysis from major clinical trials on OAC therapy in AF assessing cancer patients is shown in Table 2. In a subanalysis of the ARISTOTLE trial, the safety and efficacy of apixaban *versus* warfarin were comparable between patients with and without active cancer.^85^ Interestingly, cancer patients derived a greater benefit from apixaban therapy for the composite endpoint of stroke/systemic embolism, myocardial infarction (MI) and death. These results were replicated in an analysis of 1,153 patients initially included in the ENGAGE AF-TIMI 48 trial, who developed new or recurrent malignancy over a median follow-up of 495 days.^86^ Overall, the efficacy and safety profile of edoxaban in relation to warfarin were preserved.

**Table 2:** NOACs versus Warfarin for stroke prevention in patients with atrial fibrillation

NOAC	Primary Efficacy endpoint vs. Warfarin RR [95% CI]		Primary Safety endpoint * vs. Warfarin RR [95% CI]	
	General population **	Cancer ***	General population **	Cancer ***
Dabigatran	0.91 [0.53-0.82] †	0.14 [0.03 - 0.57] §	0.93 [0.81-1.07] †	0.23 [0.07-0.74] §
Rivaroxaban	0.79 [0.66-0.96]	0.52 [0.22-1.21]	1.03 [0.96-1.11]	1.09 [0.82-1.44] ††
Apixaban	0.79 [0.66-0.95]	1.09 [0.56-2.26]	0.69 [0.60-0.80]	0.80 [0.56-1.14] ††
Edoxaban	0.79 [0.63-0.99] ‡	0.60 [0.31-1.15] ‡	0.87 [0.73-1.04] ‡	0.98 [0.69–1.40] ‡

* Major bleeding results, unless otherwise specified.

** Data from landmark RCTs.

*** Data from post-hoc subanalysis or observational studies.

§ Results from an observational retrospective study, which included 140 patients on Dabigatran, and counted two ischemic strokes and three major bleeding events in this study arm (Kim K, et al. 2018].

† The results for Dabigatran 150mg dosage are presented.

†† Major or clinically relevant nonmajor bleeding events.

‡ The results for Edoxaban 60mg dosage are presented.

In a recently published meta-analysis comprising over 20,000 patients with AF and cancer undergoing OAC, NOACs showed lower or similar rates of thromboembolic and bleeding events when compared with warfarin (37% stroke risk reduction, 27% major bleeding risk reduction).^87^ These results are still exploratory and should be interpreted with caution until RCTs are available. One important limitation concerns the limited data about cancer staging, which might have led to uncontrolled confounding factors if the type of OACs (NOACs vs AVK) varied by cancer staging. Furthermore, patients with greater disease severity (i.e. those with reduced life expectancy) were indirectly precluded by the analysis, as they were excluded by the numerous included studies.

The individualized assessment of thrombotic and bleeding risk profile, comorbidities, and expected drug-to-drug interactions in each patient remains crucial, either before the OAC strategy initiation, when evaluating the need for dose adjustment or scheme modification, or even therapy discontinuation.

Balancing thrombotic and/or bleeding risk remains particularly challenging in specific scenarios, according to comorbidities, tumor location, staging, and cancer-related therapies, some of which are addressed in Figure 3. Although, at present, there are no data to guide the choice of specific anticoagulants in most of these extreme scenarios, refraining from using rivaroxaban, dabigatran or edoxaban in gastro-intestinal cancer patients with high bleeding risk seems advisable.

### LAA closure

Left atrial appendage (LAA) percutaneous closure was non-inferior to warfarin for the prevention of thromboembolic events and may be considered for those patients at the highest stroke risk who have contraindication for anticoagulation.^88^ The OAC is not even necessary post-procedure, as dual antiplatelet therapy in the first 6 months showed to be equally safe.^89^ It is noteworthy to remind that patients with either thrombocytopenia (platelet count <100.000) or anemia (hemoglobin <10g/dL) were excluded from major trials validating its use.

### Heart rate and rhythm control specificities in cancer patients

For symptomatic control, a strategy of either heart rate control (rate control) or sinus rhythm restoration and maintenance (rhythm control) may be reasonable. The patient’s age and functional status, comorbidities, AF duration, and predicted drug-drug interactions with rate-controlling and anti-arrhythmic drugs, are valuable aspects when deciding between the two strategies.

New-onset AF may arise in the context of systemic, infectious, metabolic, and/or endocrine disorders, and their correction may be enough to restore sinus rhythm.

Apart from these scenarios, in hemodynamically stable AF with >48h duration a rate control strategy is usually the first approach. Landmark RCT evidence showing lack of benefit with a rhythm control strategy and a lower potential for drug interactions with rate-controlling drugs have recently been questioned.^90,91^ A lenient rate control strategy is advised, with a resting heart rate objective of 100-110bpm.^92^ For this purpose, non-dihydropyridine calcium-channel blockers (diltiazem, verapamil) and digoxin carry the highest risk for relevant drug interactions with cancer treatments and beta-blockers not significantly metabolized by liver enzymes (atenolol, nadolol) may be preferred.

Antiarrhythmics have a narrow safety profile, and when choosing an antiarrhythmic agent, attention must be given to severe interactions with cancer drugs. Even in patients submitted to planned electric cardioversion for this purpose, antiarrhythmic drugs may increase the likelihood of sinus rhythm maintenance. Amiodarone is both a major CYP3A substrate and an inhibitor of P-glycoprotein and should be use with caution, when strictly necessary. Alternative antiarrhythmics in patients without structural heart disease (SHD) are sotalol, flecainide and propafenone. Mexiletine (class Ib antiarrhythmic) may be considered in those with SHD.

Data from the ORBIT-AF registry shows a 4% prevalence of prior catheter ablation procedure in AF patients with a history of cancer.^78^ There is no information on whether these procedures took place before or after the cancer diagnosis. Patients with a history of cancer were less likely to have been submitted to catheter ablation of AF, when compared with those without cancer history.

The procedure has good long-term results in experienced hands, with low complication rates. Cancer patients with a perceived life expectancy > 12 months would theoretically be plausible candidates, aiming for symptomatic and/or prognostic benefit.

### Drug-drug interactions

Although fewer food and drug-drug interactions are expected with NOAC use when compared with warfarin, some pharmacokinetic considerations have clinical relevance. A gut transporter, P-glycoprotein (P-gp), is responsible for gastrointestinal re-secretion of all NOACs. P-gp is also involved in NOAC renal secretion. Predictably, strong P-gp inhibitors result in increased NOAC plasma levels.

Cytochrome P450 3A4 (CYP3A4) enzymatic pathways are a critical step in the hepatic clearance of rivaroxaban and apixaban. Strong CYP3A4 inhibitors will potentially increase plasma levels of these drugs.

As a rule of thumb, strong inhibitors of both P-gp and CYP3A4 are not recommended in combination with NOACs. On the other hand, strong inducers of both P-gp and CY3A4, resulting in low NOAC plasma levels, may compromise treatment efficacy. Detailed drug-drug interactions and hazardous combinations have been detailed elsewhere.^93,94^

When the avoidance of severe drug-drug interaction compromises anti-cancer therapeutics efficacy, low-molecular weight heparins (LMWHs) may be considered as an alternative.

Pharmacodynamic considerations include not only the increased hemorrhagic risk with simultaneous antiplatelet therapy (e.g. in patients with acute coronary syndromes), but also the concomitant treatment with chemotherapeutic agents with antithrombotic activity. Individual assessment of thrombotic and hemorrhagic risk is advised.

### Renal and Hepatic dose adjustments

In general, NOAC use is not advised in stage V chronic kidney disease (CKD) (creatinine clearance <15mL/min/m^2^). Apixaban is considered a reasonable alternative to warfarin in these patients, according to some recommendations,^1,95^ but the supporting evidence is still weak. Patients with stage IV CKD (CrCl between 15 and 30 mL/min/m^2^) may be treated with a reduced-dose regimen of rivaroxaban, apixaban, or edoxaban. Stage III CKD (CrCl 30-60ml/min/m^2^) generally mandates NOAC dose adjustment, taking into account the patient’s characteristics affecting the drug pharmacokinetics (i.e., age and weight).

All NOACs remain contra-indicated in end-stage hepatic disease (Child-Turcotte-Pugh C cirrhosis), due to lack of data. Rivaroxaban should also be avoided in those with Child B liver cirrhosis.^93^

### Thrombocytopenia

Cancer patients with thrombocytopenia have increased bleeding risk, remaining at increased risk for thrombotic complications. To date, no robust data have emerged on which anticoagulation strategy should be pursued in this challenging scenario. It has been proposed either a strategy of platelet transfusion, or dose-modified anticoagulation regimen with LMWHs in those with severe thrombocytopenia (platelet count <50 x 109/L).^94,96^ Some causes of thrombocytopenia involving immune-mediated mechanisms are characterized by a prominent thrombotic, as well as hemorrhagic, risk. That said, there is no consensus on a lower limit of platelet count when considering anticoagulation, as this is dictated by the clinical scenario and the prevailing risk.

### Risk factor modification

Risk factor modification is crucial in AF prevention and recurrence avoidance. This includes weight loss, diabetes treatment, arterial hypertension control, sleep apnea identification and treatment, correction of thyroid dysfunction, smoke cessation, alcohol consumption avoidance, and treatment of any underlying structural / ischemic heart disease.

### Future directions

#### AF prevention

Several interventions, focusing on lifestyle and risk factor modification, prompted a significant reduction in AF burden in the general population. These include weight loss in obese patients, optimal glycemic control in DM patients, hypertension and dyslipidemia management, obstructive sleep apnea identification and treatment, smoking cessation, and alcohol consumption reduction.^97^ The extent to which cancer patients derive the same benefit with these interventions remains to be determined, but the high burden of classical cardiovascular risk factors in this population argues in favor of these interventions. Moderate aerobic exercise training is safe and provides QoL and cardiovascular benefit in cancer patients.^98^ Those integrating Cardio-Oncology rehabilitation programs experience fewer cancer therapeutic-related adverse events.^99^

#### AF diagnosis

Artificial intelligence-based algorithms for the identification of subtle ECG changes associated with future AF development (e.g. LA enlargement, Bayés Syndrome)^100^ may prove useful to identify patients who might benefit the most from AF screening. The same is true concerning echocardiographic parameters of LA dimensions and strain^101^ and/or LV systo-diastolic function.^102^ Cardiac magnetic resonance, allowing atrial morpho-functional characterization, may also become a crucial tool in early recognition of “fibrotic atrial cardiomyopathy”, which is associated with incident and recurrent AF.^103^ Genome-wide association studies (GWAS) have found several variants of atrial structural genes to be associated with AF development.^104^ Also, OMIC sciences may help refine our knowledge of the biological processes underlying incident AF, perhaps helping clinicians in its early identification and treatment.

Risk stratification models exist for myocardial toxicity and overt heart failure development, according to chemotherapeutic classes.^105^ New-onset AF may be the object of such baseline risk stratification tools in the future. This could help clinicians to better identify those patients who might benefit the most from AF screening.

The effectiveness of AF screening in cancer patients, concerning the prevention of major adverse cardio and cerebrovascular events, must be addressed in adequately powered prospective studies. The growing availability of user-friendly devices and apps, with potential for long-term screening in a large number of patients, may boost this research field.

#### AF management

Whether AF ablation carries a similar prognostic benefit in cancer patients with HFrEF, as demonstrated in the general population is currently unknown. Evidence from randomized clinical trials on NOAC use for stroke prevention in cancer patients with AF (compared with either VKA or LMWH) is also an important gap to be filled in the years to come.

## Conclusion

Cardio-oncology clinics have allowed many cancer therapeutics-related cardiotoxicity events to be prevented, early recognized and optimally managed.

Despite the high frequency of AF in patients with active malignancy, this condition remains an under-recognized comorbidity in these patients. Its frequent paroxysmal nature, together with slack screening programs, may perpetuate this situation.

AF screening in cancer patients may have a role in early AF recognition and thromboembolic event prevention, through the timely prescription of anticoagulant therapy in individuals at risk. The best screening strategy and the optimal device to improve the yield of such screening programs are yet to be established.

In the future, clinical, genetic, analytical, electrocardiographic, and echocardiographic parameters may help to stratify the risk of subsequent AF development, thereby helping in the selection of patients who deserve more stringent screening protocols.

These challenging patients, simultaneously at higher thrombotic and hemorrhagic risk, deserve dedicated clinical trials. The prognostic impact of interventions aiming at the correction of underlying structural or functional heart disease, and the optimal anticoagulant regimen, require further investigation.

Multidisciplinary Cardio-Oncology teams are at a privileged position to carry on this mission, as they warrant a truly holistic approach to these challenging patients.
